# Input-Dependent Frequency Modulation of Cortical Gamma Oscillations Shapes Spatial Synchronization and Enables Phase Coding

**DOI:** 10.1371/journal.pcbi.1004072

**Published:** 2015-02-13

**Authors:** Eric Lowet, Mark Roberts, Avgis Hadjipapas, Alina Peter, Jan van der Eerden, Peter De Weerd

**Affiliations:** 1 Psychology and Neuroscience, Maastricht University, Maastricht, The Netherlands; 2 Donders Institute for Brain, Cognition and Behaviour, Radboud University Nijmegen, Nijmegen, The Netherlands; 3 University of Nicosia Medical School, University of Nicosia, Cyprus; 4 St George’s University of London, London, United Kingdom; 5 Ernst Strüngmann Institute (ESI) for Neuroscience in Cooperation with Max Planck Society, Frankfurt, Germany; 6 International Max Planck Research School for Neural Circuits, Frankfurt, Germany; University of Pittsburgh, United States of America

## Abstract

Fine-scale temporal organization of cortical activity in the gamma range (∼25–80Hz) may play a significant role in information processing, for example by neural grouping (‘binding’) and phase coding. Recent experimental studies have shown that the precise frequency of gamma oscillations varies with input drive (e.g. visual contrast) and that it can differ among nearby cortical locations. This has challenged theories assuming widespread gamma synchronization at a fixed common frequency. In the present study, we investigated which principles govern gamma synchronization in the presence of input-dependent frequency modulations and whether they are detrimental for meaningful input-dependent gamma-mediated temporal organization. To this aim, we constructed a biophysically realistic excitatory-inhibitory network able to express different oscillation frequencies at nearby spatial locations. Similarly to cortical networks, the model was topographically organized with spatially local connectivity and spatially-varying input drive. We analyzed gamma synchronization with respect to phase-locking, phase-relations and frequency differences, and quantified the stimulus-related information represented by gamma phase and frequency. By stepwise simplification of our models, we found that the gamma-mediated temporal organization could be reduced to basic synchronization principles of weakly coupled oscillators, where input drive determines the intrinsic (natural) frequency of oscillators. The gamma phase-locking, the precise phase relation and the emergent (measurable) frequencies were determined by two principal factors: the detuning (intrinsic frequency difference, i.e. local input difference) and the coupling strength. In addition to frequency coding, gamma phase contained complementary stimulus information. Crucially, the phase code reflected input differences, but not the absolute input level. This property of relative input-to-phase conversion, contrasting with latency codes or slower oscillation phase codes, may resolve conflicting experimental observations on gamma phase coding. Our modeling results offer clear testable experimental predictions. We conclude that input-dependency of gamma frequencies could be essential rather than detrimental for meaningful gamma-mediated temporal organization of cortical activity.

## Author Summary

Almost 350 years ago the physicist and polymath Christiaan Huygens first observed the synchronization between two pendulum clocks attached to a common support. Since then synchronization has been recognized as a universal phenomenon from astronomy to biology. The phase-locking (synchrony) and the phase-relation between the two pendulums are determined by two principal forces: the synchronization force exerted over the connection and the tendency to desynchronize due to frequency (speed) differences. We propose that gamma synchronization (25–80Hz) among oscillating cortical neurons in the brain can be understood according to the same principles—like a field of many connected pendula—with the critical addition that input changes the frequency of gamma oscillations, as shown by recent experimental studies. It has been assumed that input-dependent changes in oscillation frequency are detrimental for a meaningful role of gamma synchronization in neural processing. To the contrary, our theoretical analysis demonstrates that because input *can* change the frequency of the oscillation, phase-locking and phase-relations among neurons relate systematically to input. By analogy, it is because a local push to a pendulum will change its frequency, that resulting changes in phase-locking and phase-relation among the pendula can be used to derive the external force applied.

## Introduction

How the millions of neurons in the brain are coordinated to permit meaningful computations is one of the fundamental questions of neuroscience. Spike synchrony and relative spike timing play important roles in dynamically coordinating neural activity [1–7] with substantial impact on neuronal function [8–12]. Synchronization often goes hand in hand with neural oscillations, of which gamma-band oscillations (∼25–80Hz) have received broad attention [13–15]. Gamma oscillations occur in various brain regions and species [13–16]. Gamma oscillations arise locally from mainly direct interactions between inhibitory and excitatory neurons [14,15,17,18]. Modulations of gamma oscillation properties (power, frequency) have been found for various cognitive functions including perception [19–21], attention [22–24], working memory [23] as well as in psychiatric disorders like psychosis [25,26] and ADHD [27,28]. At the neuronal level, different roles (that are not mutually exclusive) have been suggested; they include neural grouping by phase-locking within [21,29–31] and between cortical areas [13,32,33], phase coding [15,18,34–37], neuronal plasticity [38,39], gain control [18] and normalization [40].

However, the role of gamma oscillations in neural computation is controversial, with judgments ranging from fundamental [13,14,21] to epiphenomenal [41–43]. Experimental studies have given conflicting evidence on the role of gamma phase coding of input drive. For example, Vinck et al. [34] have shown that visual cortical neurons receiving different input drive (through varying stimulus orientation) can exhibit reliable spike timing differences in the gamma oscillation range. However, Montemurro et al. [44] using natural stimuli could not find any contribution of visual cortical gamma phase to the encoding of the input. Similarly, McLelland and Paulsen [45] did not find a rate-to-phase transform for gamma oscillations, which would assign a specific level of input to a specific phase of gamma. Moreover, although various experimental studies [29,31,43] have shown input(stimulus)-dependent changes in gamma synchronization, theoretical models [21,46,47] have fallen short in convincingly including the local and variable nature of gamma oscillations. For example, the dependence of gamma oscillation frequency on stimulus attributes (e.g. visual contrast [33,42,43]) as well as the limited spread of gamma phase-locking over cortical distance [48,49] are seen as conflicting with a functional role of gamma oscillations in neural processing [40–42,50].

Here, we used computational modeling techniques to develop a deeper understanding of input-dependent cortical gamma synchronization. We focused on the underlying organization principles of phase and frequency coding of input drive and its relation to spatial synchronization and network connectivity. Mathematically, the synchronization principles of interacting limit-cycle oscillators (and other types, [51]) is well understood [52–54]. In particular, the theory of weakly coupled oscillators (TWCO) (see [55] for review) has proven to be useful and has been applied in many scientific domains, including neuroscience [52–54,56–60]. In TWCO the phase of an oscillator (neuron, group of neurons) is defined by an intrinsic (natural) frequency. The interaction with other oscillators is characterized by the phase response curve (PRC, [61]) which defines how the phase is modified by the interaction. Crucially, the phase-locking between oscillators depends on the intrinsic frequency difference (described as the detuning level) as well as interaction strength (or coupling strength), defining the so called Arnold tongues (region of synchronization defined by the interplay of detuning and coupling) [18,55,62]. Note that in TWCO, the coupling strength is considered to be ‘weak’, meaning that the interactions among oscillators mainly change the phases but not the oscillation amplitudes.

A few prior studies have concretely considered TWCO for explaining input-dependent cortical gamma synchronization [18,53,54,63–65]. Of most relevance here, Tiesinga and Sejnowski [18] first used TWCO in a biophysically realistic gamma network for explaining gamma phase coding in visual cortex [34]. Several interconnected pyramidal-interneuron-gamma networks (PING) synchronized on a common frequency, despite receiving different levels of input currents, and converted input differences into phase-differences.

Despite these important advances, the organization principles of gamma oscillations in cortical networks, characterized by local synchrony and input-dependent oscillation frequencies over cortical space, have so far not been systematically investigated. In particular, so far, the theoretical principles that determine the phase-locking and phase-relationship among interconnected gamma-oscillating neurons receiving different input levels are as yet not well understood. In the present study, we study whether TWCO may offer a framework to describe these organization principles. Moreover, it is currently poorly understood how much information about the stimulus input is encoded in the phase-relation and frequency differences among neurons. To answer these questions, we investigated spatially-defined excitatory-inhibitory (PING) networks that were, similarly to cortical networks, topographically organized with spatially local connectivity and spatially-varying input drive. The network exhibited local spatial synchrony and could express different gamma frequencies at different locations at the same time. The gamma frequency ranges were set to match our own observations in awake monkey V1. We used several networks varying in size and complexity. In all of them, we observed that phase-locking, phase-relations and frequency differences among neurons resulted from an interplay between detuning (Δintrinsic frequency) and coupling strength, in accord with TWCO and the Arnold tongue. Critical for the behavior was the property of gamma oscillations to shift their preferred frequency with input drive. Phase and frequency coding of input was largely complementary in accordance to the Arnold tongue concept, whereby conditions inside the Arnold tongue lead to phase coding, and conditions outside lead to frequency coding. A combined frequency and phase coding could best reconstruct the stimulus input. Importantly, the Arnold-tongue based phase coding implied a relative Δrate-to-phase transform and therefore gamma phase told little about absolute input levels. Our work has clear theoretical implications leading to experimentally testable predictions that are elaborated in the Discussion.

## Methods

### Experimental procedures

**Figure 1:**
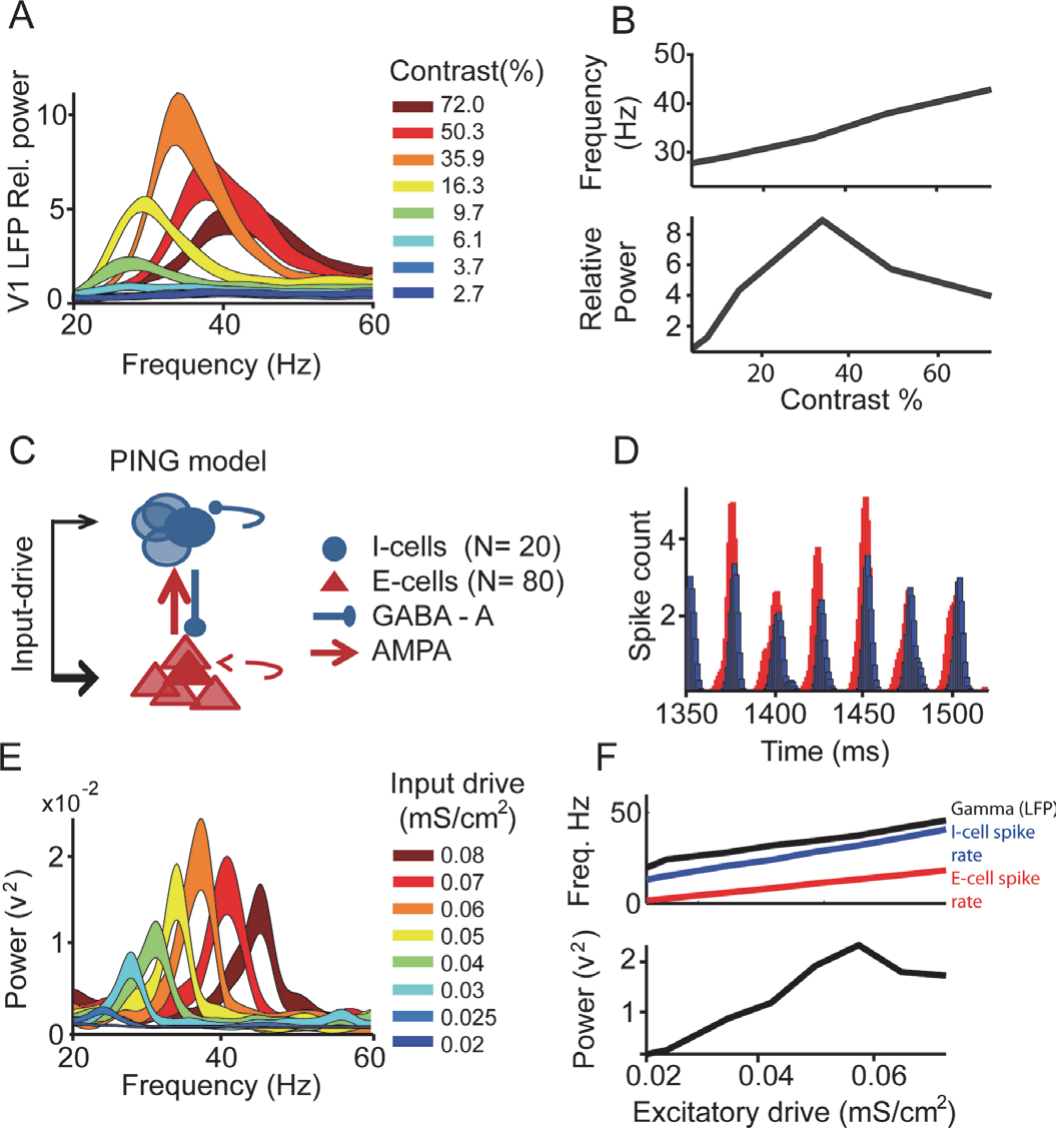
Luminance contrast and input-drive dependent gamma oscillation frequency. (A) V1 LFP mean relative power spectra (20–60Hz, line thickness represents ±1 SEM) during presentation of square wave gratings of 8 different luminance contrasts (line color), mean data from Monkey S [8].(B) Gamma band peak frequency (top) and power (bottom) as a function of contrast (only the 6 highest contrast conditions). (C) Schematic architecture of the pyramidal (red)—interneuron (blue) gamma network (PING). (D) Example time period of population spike histogram (2ms bins) during steady excitatory drive input (0.06 mS/cm^2^). Spikes of the excitatory (red) neurons occurred earlier than from inhibitory (blue) neurons within a gamma cycle. (E) The absolute power spectra for different input excitation levels (mimicking contrast). (F) Quantification of (upper panel) gamma frequency (black), I-cell spike rate (blue), E-cell spike rate (red) and (lower panel) gamma power as a function of excitatory input drive.

Experimental observations in Fig. 1 and associated methods of data collection shown have been described in a previous publication [33]. We show here only data from monkey S V1 for illustration purposes only. We re-analyzed the LFP spectra obtained during stimulation (Stim) with static square-wave grating (2 cycles per degree), using a multi-taper method with discrete prolate spheroid sequences for frequencies 20 to 60Hz (smoothing ± 3Hz) in non-overlapping 500ms windows starting 350ms after stimulus onset. LFP power in the pre-stimulus baseline (Base) was calculated from the 500ms period before stimulus onset. Relative power was calculated as (Stim-Base)/Base, where Stim and Base were calculated separately after averaging over trials. In Fig. 1B the quantifications of maximum of peak gamma power as well as frequency of peak power is shown for the Michelson contrast conditions 6.1%, 9.7%,16.3%,35.9%,50.3% and 72%. The stimulus contrast conditions 2.5% and 3.7% had very low induced gamma power and no clear peak in the power spectrum and therefore not included.

### Computational resource

Intel(R) Xeon(R) CPU E5-1620 0 @3.6GHz with 16GB RAM.

### Hodgkin-Huxley network

**Figure 2:**
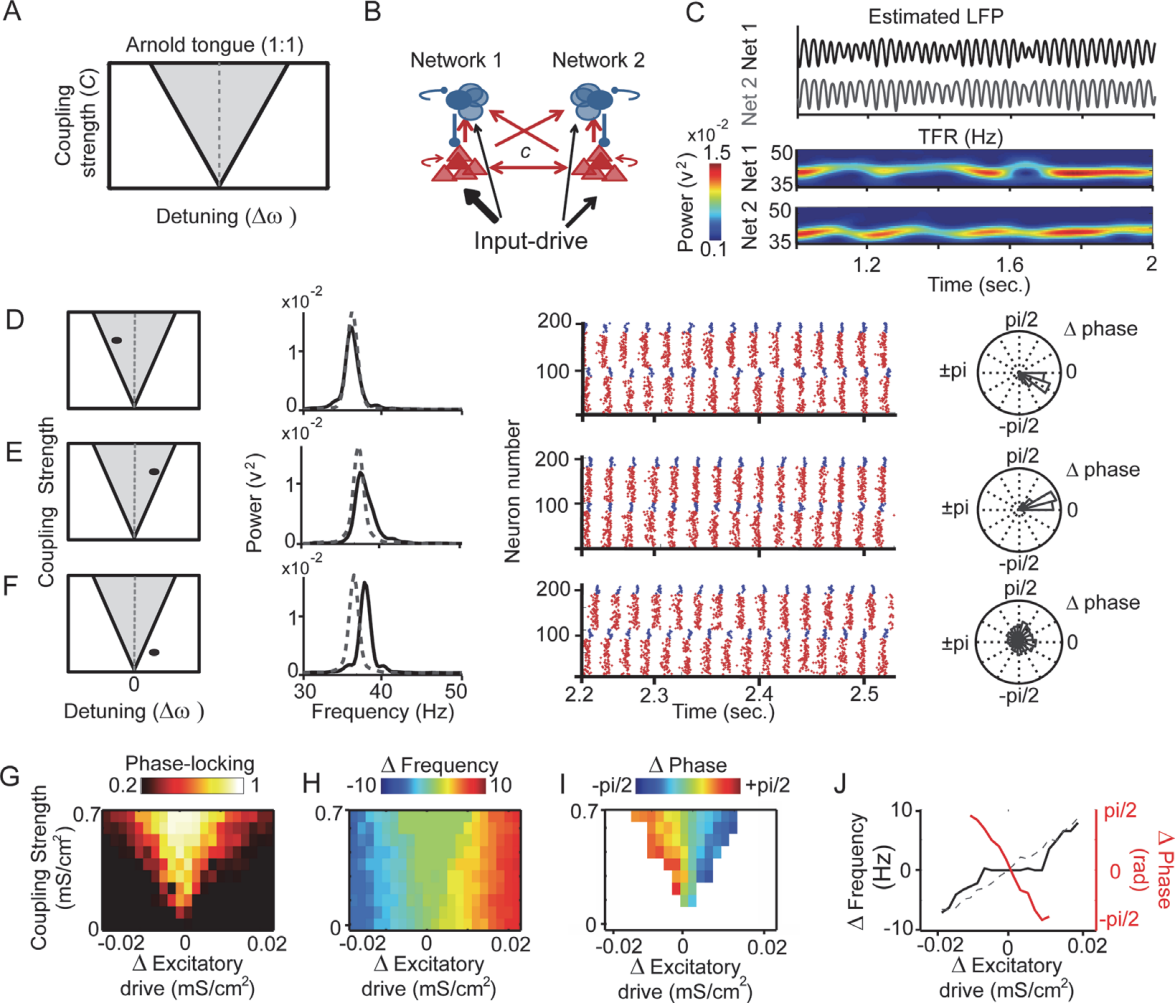
Impact of oscillation frequency on the interaction between two PING gamma networks. (A) Illustration of the Arnold tongue. The potential for two oscillators to synchronize (grey area) is positively correlated with coupling strength and negatively correlated with the difference in intrinsic frequency. (B) The main structure of the two coupled PING gamma networks. Excitatory drive difference (detuning, [Δω) and the coupling strength *C* between the two networks were modulated. (C) Example simulation output from the networks, upper) smoothed LFP signal, arbitrary scaling, lower) time-frequency representation. (D–F) Example simulations with different network parameters: Left to Right: Arnold tongue: Parameters Δω and Coupling Strength are indicated as black dot in Arnold tongue diagram. Power spectra, shown in black line for simulated LFP for network 1 and in dashed grey line for network 2. Population raster plots shown for simulation window [2.2sec to 2.55sec]: network 1 = neuron 1–100, network 2 = neurons 101–200. E-cells spikes are indicated by red dots, I-cells spikes by blue dots. Polar plot of phase difference are shown to the right. Bar length indicates percentage of time (from 5s trial) within each phase bin. G-I) Reconstruction of Arnold Tongue when manipulating coupling strength and detuning (detuning = input-drive_network1_—input-drive_network2_). Arnold Tongue corresponds to a region with strong phase locking (G, bright colors), common emergent frequency (H, green color), and systematic phase difference (I, color-coded) between coupled networks, Network 1 lagged in phase compared to network 2 (red in I) when network 1 received a lower drive. Conversely, network 1 had a leading phase relationship with network 2 (blue in I) when network 1 received the higher excitatory drive Δ phase is only shown for conditions of substantial phase locking (>∼0.3) (J) Overlaid representation of emergent frequency difference (black line), intrinsic frequency difference (dashed line) and phase difference (red line) for simulations with inter-network excitatory connections of 0.6 mS/cm^2^.

**Figure 3:**
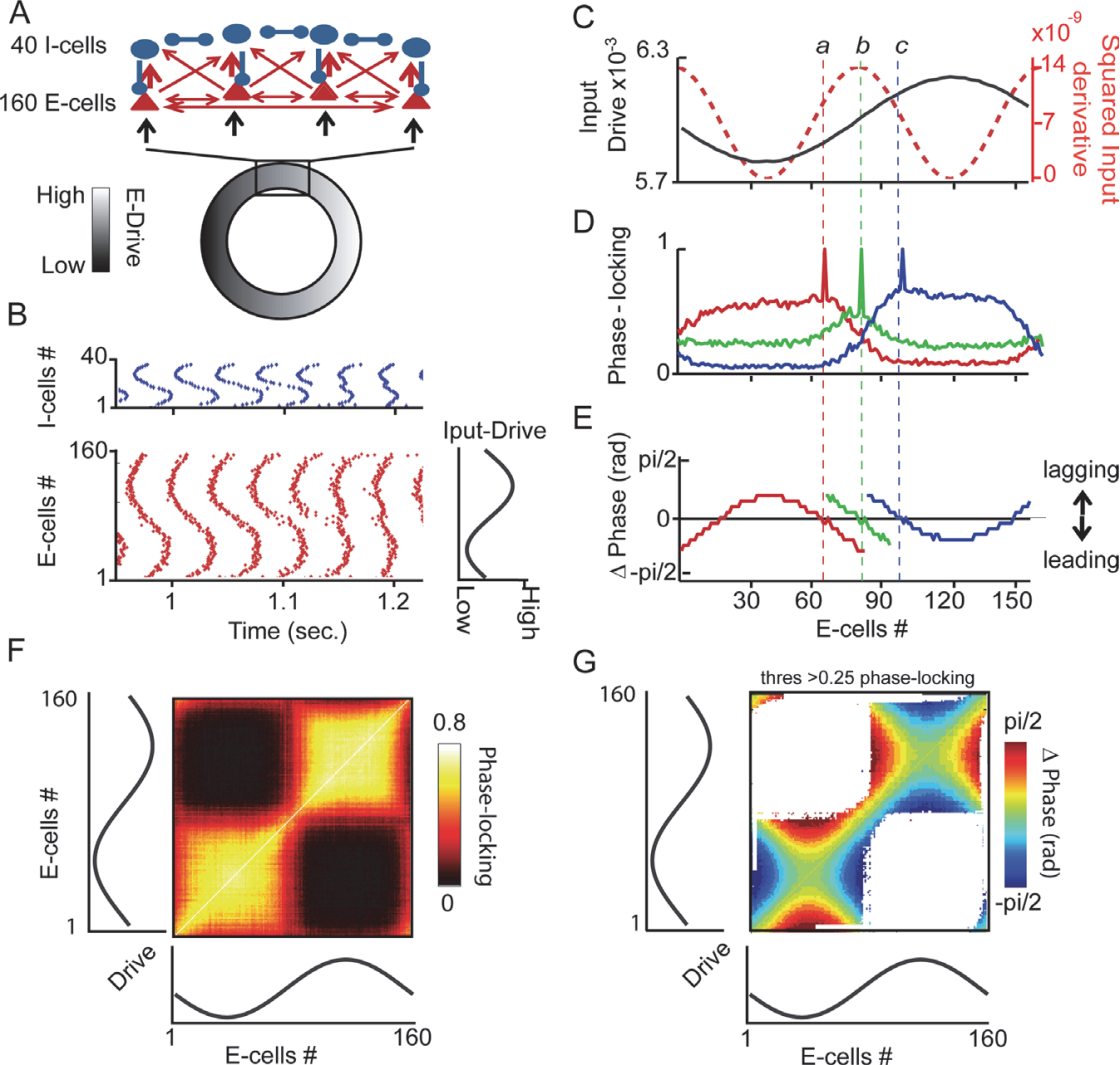
Assembly formation and complementary rate/phase code. (A) The overall network ring structure of the PING model with nearest-neighbor connections (B) Example of a simulation population spike raster output (top I-cells, bottom E-cells), variation in E-cell excitatory drive is indicated to the right.(C to E) Detailed results for three example E-cells ***a*, *b*** and ***c***. (C) Location of each example E-cell along the ring structure is indicated by the level of input-drive (black) as well as the squared derivative of input (red). (D) Phase-locking values between each example E-cell and all other E-cells (estimated by cross-correlation peak). (E) Phase difference between example E-cell and all other E-cells with phase-locking threshold >0.25 (for illustration, see Methods). (F) Matrix showing phase-locking between all possible pairs of E-cell pairs, location in the ring is indicated by the level of input-drive, as in C. (G) Phase difference between all possible E-cell pairs with same phase-locking threshold as above. Blue indicates that the X-axis neuron leads the Y-axis neuron, red indicates the reverse.

Minimal single-compartment Hodgkin-Huxley models [66] were used to construct E-cells (regular-spiking excitatory neurons, RS) and I-cells (fast-spiking inhibitory interneurons, FS). For the network simulations shown in Fig. 1 and Fig. 2 the networks consisted of 80 E-cells and 20 I-cells. For Fig. 3 the network consisted of 160 E-cells and 40 I-cells neurons. The E-cells in Fig. 3 had particularly high firing rate matching the network gamma frequencies. This was done to increase oscillatory stability of the small network which was limited in size due to computational constraints. We use Izhikevich-type neurons [67] for replicating our findings in larger E-and I-cells networks (see S1 Text). In Fig. 3 neurons were ordered along a ring to avoid network border effects (continuous connectivity). Numerical simulations were computed using a variable step size Runge-Kutta method of order 8 according to the Dormand and Prince algorithm [68]. The simulation code was written in FORTRAN95. Analysis of the simulation output was performed with Matlab (MathWorks, R2012b).

### Model summary

Regular-spiking (RS) E-cell:

$$C_{m}\frac{dV}{dt}=-g_{\mathrm{Leak}}\left(V-E_{\mathrm{Leak}}\right)-I_{\mathrm{Na}}-I_{K}\;-I_{M}$$

Fast-spiking (FS) I-cell:

$$C_{m}\frac{dV}{dt}=-g_{\mathrm{Leak}}\left(V-E_{\mathrm{Leak}}\right)-I_{\mathrm{Na}}-I_{K}$$

The leakage reversal potential and conductance were *E_Leak_* = −70*mV* and *g_Leak_* = 0.0205*mS* / *cm* ^2^ for E-cells and *g_Leak_* = 0.015*mS* / *cm* ^2^ for I-cells. The membrane capacitance was *C_m_* = 1 mF/cm^2^. All kinetic parameters were according to a temperature of 36ºC using standard conductance equations.

### Conductances

Sodium current *I_Na_*:

RS: $E_{NaÊ}=50Ê\;mV\;,\;{\bar{g}}_{Na}=50mS/cm^{2}\;V_{T}=\;-61.5mV$

FS: $E_{Na}=50\;\mathrm{mV}\;,\;{\bar{g}}_{Na}=46\mathrm{mS}/cm^{2}\;V_{T}=-61.84mV$

$$I_{Na}={\bar{g}}_{Na}\;m^{3}\;h\left(V-E_{Na}\right)$$

$$\frac{dm}{dt}=\alpha_{m}\left(V\right)\left(1-m\right)-\beta_{m}\left(V\right)m$$

$$\frac{dh}{dt}=\alpha_{h}\left(V\right)\left(1-h\right)-\beta_{h}\left(V\right)h$$

$$\alpha_{m}=\frac{-0.32\left(V-V_{T}-13\right)}{\exp\left[-\frac{V-V_{T}-13}{4}\right]-1}$$

$$\beta_{m}=\frac{0.28\left(V-V_{T}-40\right)}{\exp\left[-\frac{V-V_{T}-40}{5}\right]-1}$$

$$\alpha_{h}=0.128\;\exp[-\frac{V-\;V_{T}-17}{18}]$$

$$\beta_{h}=\frac{4}{1+\exp\left[-\frac{V-V_{T}-40}{5}\right]}$$

Delayed-rectifier potassium current *I_Kd_*:

RS: $E_{Kd}=-90\;mV\;,\;{\overset{‒}{g}}_{Kd}=4.8mS/cm^{2}\;V_{T}=-61.5mV$

FS: $E_{Kd}=-90\;mV\;,\;{\bar{g}}_{Kd}=5.1\;mS/cm^{2}\;V_{T}=-61.84mV$

$$I_{Kd}={\bar{g}}_{Kd}\;n^{4}\;(V-E_{k})$$

$$\frac{dn}{dt}=\alpha_{n}\left(V\right)\left(1-n\right)-\beta_{n}\left(V\right)n$$

$$\alpha_{n}=\frac{-0.032\left(V-V_{T}-15\right)}{\exp\left[-\frac{V-V_{T}-15}{5}\right]-1}$$

$$\beta_{n}=0.5\exp\left[-\frac{V-V_{T}-10}{40}\right]$$

Slow non-inactivating potassium current *I_M_*:

RS: $E_{Km}=-90\;\mathrm{mV}\;,\;{\bar{g}}_{Km}=0.15\;\mathrm{mS}/cm^{2}\;V_{T}=-61.5\mathrm{mV}\;,\;\tau_{\max}\;=1123.5\mathrm{ms}$

$$I_{M}={\bar{g}}_{M}\;p\;(V-E_{k})$$

$$\frac{dp}{dt}=(p_{\infty }(V)-p)/\tau_{p(V)}$$

$$p_{\infty }(V)=\frac{1}{1+\exp\left[-\frac{V+35}{10}\right]}$$

$$\tau_{p}(V)=\frac{\tau_{max}}{3.3\exp\left[\frac{V+35}{20}\right]+\exp\left[-\frac{V+35}{20}\right]}$$

### Synapses

Synaptic excitatory AMPA and inhibitory GABA-A potentials were modeled based on [69]. The synaptic current into neuron α was:

$$I_{\mathrm{syn},\alpha }=G_{\mathrm{in},\alpha }\left(V_{\alpha }-V_{\mathrm{in}}\right)+G_{\mathrm{ex},\alpha }\left(V_{\alpha }-V_{\mathrm{ex}}\right)$$

Here, the total synaptic conductance from inhibitory presynaptic neurons was:

$$G_{\mathrm{in},\alpha }=\sum_{\beta \;\mathrm{in}}g_{\beta \to \alpha }s_{\beta }\;$$

The expression for the excitatory synaptic conductance was of the same form. It was assumed that the dynamics of all synapses of a given (presynaptic) neuron were perfectly synchronized. Hence a synaptic gate, though physically located on the post-synaptic neuron α, followed the potential *V_β_* of the pre-synaptic neuron β with parameters shown in Table 1. For maximum conductance values *g_β→α_* see above.

$$\frac{{\mathrm{ds}}_{\beta }}{\mathrm{dt}}=a_{\beta }\left(1+\tanh\left(\frac{V_{\beta }}{4}\right)\right)\left(1-s_{\beta }\right)-\frac{s_{\beta }}{\tau_{\beta }}$$

**Table 1:** The synaptic parameter values for AMPA and GABA-A.

	**Reversal potentials (mV)**	**α_β_(ms^−1^)**	**τ_β_(ms)**
**AMPA**	V_in_ = 0	1	2.4
**GABA-A**	V_ex_ = −80	2	20

From left to right: The reversal potential (in mV), the rising time constant α_β_ and decay time constant τ_β_ are shown for AMPA (middle row) and GABA-A (bottom row).

### Connectivity

For Fig. 1–3 the network connectivity parameters (in mS/cm^2^) are listed in Table 2. The connectivity matrix network was based on the number of neighbor connections m. For example, m = 8 meant that a neuron connected to the closest 8 other neurons with unit connection strength. For E-I connections, m meant that I-cell received input from m E-cells (afferent). For I-E, m meant that I-cell sent input to m E-cells (efferent). The connectivity parameters were normalized (divided) by the number of connections m. The chosen parameters are listed in Table 3. We describe effects of changing coupling parameters in S1 Fig.

**Table 2:** Connectivity number and strength of synaptic connections in the Hodgkin-Huxley networks of Fig. 1 and Fig. 2.

**Within network**	**Number of connections m**	**Connection strength g_β→α_ (mS/cm^2^)**
E → I (afferent)	20	0.6
E → E (afferent)	35	0.05
I → E (efferent)	20	0.8
I → I (efferent)	15	0.5
**Between network**		
E→E (afferent)	10	0–0.07
E→I (afferent)	10	0–0.07

Left column specifies which cell-types are connected. ‘Afferent’ means that the connection number m defines how many connections from the sending neuron population a neuron receives. ‘Efferent’ means how many connections a sending neuron has with the receiving neuron population. Middle column shows the number of connection m per neuron. The right column shows the synaptic connection strength (E→I or E→E: AMPA, I→E or I→I: GABA-A).

**Table 3:** Connectivity number and strength of synaptic connections in the ring-PING network (Fig. 3).

	**Number of connections m**	**Connection strength g_β→α_ (mS/cm^2^)**
E → I (afferent)	10	0.23
E → E (afferent)	25	0.03
I → E (efferent)	10	0.13
I → I (efferent)	4	0.1

Left column specifies which cell-types are connected. ‘Afferent’ means that the connection number m defines how many connections from the sending neuron population a neuron receives. ‘Efferent’ means how many connections a sending neuron has with the receiving neuron population. Middle column shows the number of connection m per neuron. The right column shows the synaptic connection strength (E→I or E→E: AMPA, I→E or I→I: GABA-A).

### Network input

The input to each neuron consisted of an external excitatory input plus internal excitatory and inhibitory input via network connections. The external input consisted of a train of AMPA synaptic conductance spikes (double exponentials: rising constant = 1ms, decaying constant = 5.2ms) with Poisson statistics at a rate of 800Hz (±SD = 100) and spike amplitudes of default 0.02mS/cm^2^ (±SD = 0.002). The default mean AMPA input level to each neuron was 0.01mS/cm^2^ for FS neurons. For Fig. 1 the mean amplitude of the AMPA synaptic potentials were modulated from 0.02 to 0.08mS/cm^2^ for RS by modulating the spike amplitude. In Fig. 2 the mean AMPA conductance input level was 0.06 mS/cm^2^. In Fig. 3 each neuron received a spatially specific input level depending on its position in the ring architecture. The amplitude of the sinusoidally modulated AMPA conductance was of 0.006mS/cm^2^ and the mean conductance AMPA input 0.06mS/cm^2^ and 0.055 mS/cm^2^ for S1 Fig.

### Spike detection and network signal

The voltage used as the spike detection level was −17.5mV for both E- and I-cells. The local field potential (LFP) was estimated in units of microVolt (μV) for Fig. 1 & 2 as an overall network signal. The LFP was the extracellular electrical field potential LFP = LFP (**r**_0_,t) at an electrode position **r**_0_. We treated neuron i at position **r_i_** as a point-current source I = I (**r_i_**,t) (total transmembrane current into the neuron) in a homogeneous extracellular medium with conductivity σ (1/σ = 0.3 kΩcm), taken from [70]: 0.2–0.4 kΩcm). We summed the individual neuron contributions according to the quasistatic Maxwell equations:

$$\mathrm{LFP}=\frac{1}{4\pi \sigma }\sum_{i}^{N}\frac{I\left(r_{i},t\right)}{R}$$

with $R=\left|r_{i}-r_{0}\right|$ the distance of the point source to the electrode (R = ∼1mm). The extracellular voltage signal was smoothed with a pseudo-Gaussian function (width = 4ms).

For computing local synchronous rhythmic activity of local population of neurons in S1 Fig. to show effects on noise on rhythmic population activity and rhythmic single neuron activity, we derived a local population average signal (LPA) based on the spike trains of the neurons. For each position in the network, we aggregated the spike activity of the whole network weighted by a spatially exponentially decaying function.

$$\mathrm{LP}A_{i}=\frac{1}{N}\sum_{j=1}^{N}r_{i}\exp^{-D_{i,j}/s}$$

With r_i_ being the binary spiking variable, D_i,j_ being the spatial distance between neurons (defined circularly on the ring in radians). S corresponds to the spatial decay constant which was chosen to be 0.4. The rationale of the value (similar results were observed for a large range of values) was that it was large enough to allow for sufficient aggregation to quantify oscillatory activity and spatially specific enough to reveal the spatial change of phase-locking and phase-relation. The LPA was further smoothed with a pseudo-Gaussian function (width = 4ms).

### Natural contrast images and intrinsic frequency map

We obtained natural images from the Berkeley segmentation dataset (BSDS500, [71]). We took the first 100 gray-scale natural images (comprising the Berkeley training dataset). The natural images were first resampled and squared to fit it to the 100×100 lattice. Then the local root-mean squared contrast *C* = *C* (*x_c_, y_c_*) at image position (*x_c_, y_c_*) (RMS, [72]) was computed:

$$C=\sqrt{\sum_{i=1}^{N}w_{i}\frac{{\left(L_{i}-L\right)}^{2}}{L^{2}}/\sum_{i=1}^{N}w_{i}}$$

$$w_{i}=0.5\left(\cos\left(\frac{\pi }{p}\sqrt{{\left(x_{i}-x_{c}\right)}^{2}+{\left(y_{i}-y_{c}\right)}^{2}}\right)+1\right)$$

L is luminance and **i** is the pixel index. The summation was over pixels within a patch radius p of 3 pixels. The local contrast values *C* were then transformed into intrinsic frequencies ν by approximating the experimentally observed relationship ν = 25 + 0.25*C* between gamma frequency and contrast. We defined a minimum (25 Hz) and slope of 0.25Hz per contrast value (estimated over both monkeys,[33]). Gamma power was not taken into account in the phase-oscillator model. 20 of 100 images were excluded because not enough segmentation borders (see criteria below) suitable for phase-locking analysis (minimum of > 10 per image) could be obtained.

### Phase-Oscillator model

We used a modified version of the Kuramoto model [56] as a basic model of the dynamics of a limit-cycle oscillators that has been used to investigated synchronization between coupled oscillators. The network input was set by natural images transformed into local contrast. The intrinsic frequency of each oscillator was set by the local contrast at the oscillator’s corresponding pixel. For each image, the simulation run was 10s with a time step of 2ms. Each oscillator started with a random phase. During the simulation run the phase of each oscillator was determined by an intrinsic (natural) frequency (ω), a noise term (ζ) and an interaction term describing the impact (phase response curve, PRC) by other coupled oscillators depending on the coupling constant (K).

$$\frac{d\theta_{i}}{\mathrm{dt}}=\omega_{i}+\zeta_{i}+\sum_{j=1}^{N}K_{i,j}(\sin(\theta_{j}-\theta_{i})),\;i=1\ldots N$$

The interaction term (infinitesimal PRC, [56]) was a sinusoidal function such that the coupled oscillators tended to engage in zero-phase synchrony. The coupling constant was an exponential function of distance (D) (in contrast to the all-to-all connectivity in the Kuramoto model), with a scaling constant (s = 0.4 for ring-network (radians) and s = 0.5 for 2D lattice network (pixel)) and strength (C = 0.003).

$$K_{i,j}=C\;\exp^{-D_{i,j}/s}$$

The noise term was pink noise with a power scaling exponent of 1. The strength C was scaled at a sufficient level for the model to reach near-zero coherence when oscillators were uncoupled. The noise was spatially correlated (smoothed with spatial kernel of 3 pixels), to reduce spurious phase locking over the lattice. This step eased the computation of ‘true’ phase-locking between distant clusters having very close frequencies (without the use of phase-perturbation techniques), because synchronous clusters cannot easily average out noise (correlated between members of a cluster). We also included a time-delay term as function of distance as conduction delay of cortical horizontal connections can be significant for longer cortical distances [73,74].

$$T_{i,j}={\mathrm{vD}}_{i,j}\;+\;v_{o}$$

Where the time-delay T_i,j_ was a linear function of distance (pixel units). The slope v was 0.4 and v_0_ was 2ms. T_i,j_ was then made then discrete to change in steps of 2ms (simulation time step). The inclusion of the time-delay factor was not critical for the results of the paper. Natural images and segmentations by human observers were taken from the Berkeley segmentation dataset [71]. Images were downsampled from 350×450 pixels to 100×100 pixels using the Matlab in-built ‘imresize’ function, to fit the size of the lattice model.

### Spectral power estimations

In Fig. 1 as well as S2 Fig. we used the Matlab in-built power spectral density function (psd) with multitaper estimation for estimating the power spectrum. For the time-frequency representation (TFR) in Fig. 2c we used the Matlab in-built spectrogram function (Short-time Fourier transform).

### Instantaneous phase and frequency estimations

The instantaneous phase (IP) was derived for the LFP (Fig. 1–2) or LPA (S2 Fig.) signals by taking the Hilbert-transform (HT, [75]) of the signal. The HT gives the analytical signal (complex numbers) from which the IP can be obtained by taking the argument of the complex number. The HT is well defined for signals characterized by a single oscillation (mono-component) which was the case in our simulations. The IP was the output variable of the phase-oscillator model. The instantaneous frequency (IF) was obtained by taking the derivative of the IP. IF was estimated by unwrapping the IP, then first applying smoothing (half-cycle rectangular points smoothing) followed by computing the first derivative. For the phase-oscillator model (Fig. 5–8), we could directly use the output phase-traces to compute IF. We averaged the IF estimation of each time point over the whole simulation (excluding the first 200ms) period to obtain a mean frequency. For single neurons spike trains we used the spike rate, computed as n spikes per second, as our frequency estimation.

**Figure 4:**
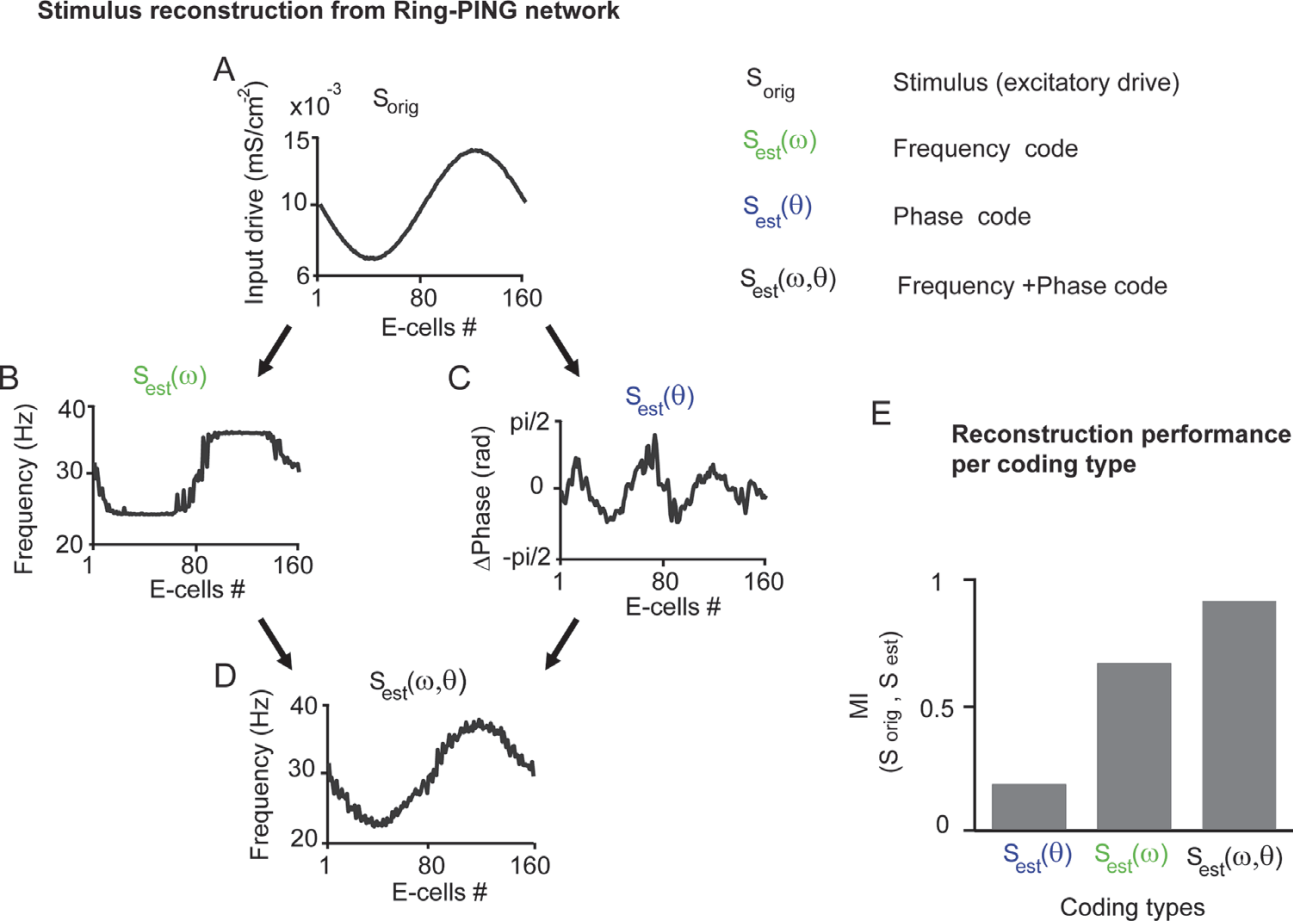
Reconstruction of stimulus input based on phase and frequency coding. See Methods for derivations of the coding schemes. A) The stimulus input S_orig_ to be reconstructed B) Reconstruction based on frequency S_est_(ω) (here E-ell rate) alone C) based phase-differences among E-cells S_est_(θ) D) based on a combined frequency and phase code S_est_(ω,θ). E) The reconstruction performance, measured by mutual information (MI), was from lowest to highest MI = 0.18 for S_est_(θ), MI = 0.65 for frequency code S_est_(ω) and MI = 0.92 for combined code S_est_(ω,θ).

**Figure 5:**
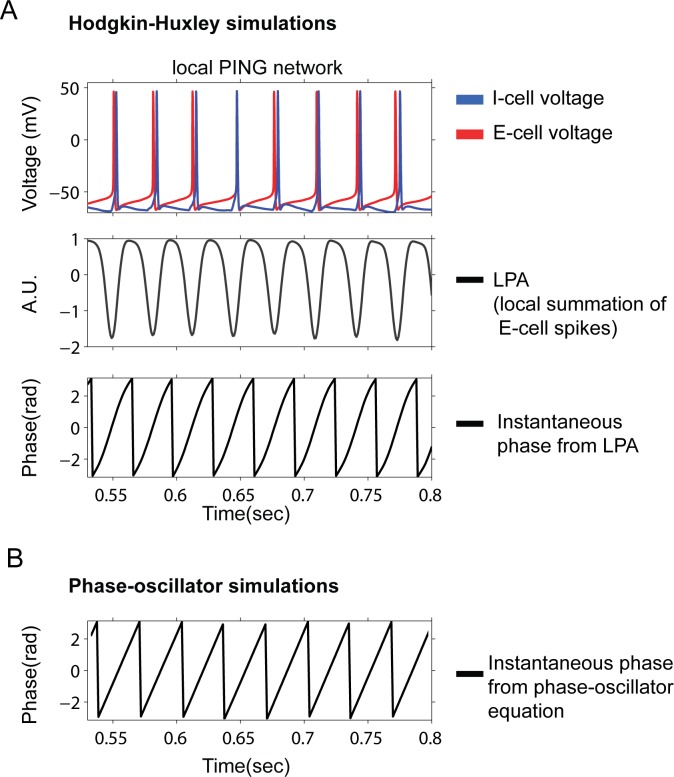
Comparison of Hodgkin-Huxley networks (HH) and phase-oscillator model. A) The voltage membrane of an E-cell and I-cell is shown as modeled by the HH-dynamical equations. The generative mechanism of PING gamma oscillation is the rhythmic interaction of E- and I-cells. In the middle, the LPA (local population average of E-cell spikes, see Methods) is shown. The fluctuations in the LPA represent the synchronous rhythmic interactions among the local population of E- and I-cells. Using the Hilbert transform, one can easily derive the instantaneous phase of the LPA as shown below. B) The instantaneous phase of a phase-oscillator is shown. In the phase-oscillator model the phase-variable is modeled directly by one simple dynamical equation (see Methods) mainly governed by the intrinsic frequency and interactions by other oscillators. Our assumption in this study is that the instantaneous phase derived from local LPA in the HH-PING network can be approximated by phase-oscillators.

### Phase-locking and phase relation estimations

**A. Based on Instantaneous phase (LFP,LPA and phase-oscillators)**

The phase relation was defined as the mean circular phase difference between two signals (averaged in the complex domain).

$$\theta_{ij}=\arg(\frac{1}{N\;}\sum_{t=1}^{N}\exp\left(i\;(\varphi_{i}-\varphi_{j)}\right))$$

with a range of [−π, π]. Arg is the argument function and *φ* is the IP. For estimating phase-locking we computed the phase-locking value (PLV, [76]). The PLV was computed by averaging the complex values with unit amplitude

$$\psi_{ij}=|\frac{1}{N}\sum_{t=1}^{N}\exp\left(i\;(\varphi_{i}-\varphi_{j)}\right)|$$

The PLV ranges from 1, corresponding to full phase consistency, to 0, corresponding to fully random.

**B. Based on spike trains**

For computing the phase-relation and locking between two neurons we applied cross-correlation.

$$CC_{ij}\left(l\right)=\frac{1}{N}\left(\sum_{n=0}^{N-1}r_{i}^{*}\left[n\right]r_{j}\left[n+l\right]\right)$$

with $r_{i}^{*}$ being the complex conjugate (*) of spike train (r) of neuron i. The cross-correlation *CC_ij_* between neuron I and j was computed with lags not exceeding +/− half mean rate (time window is assumed to be the period of the oscillation the neurons are locked to). The spike timing difference (in ms) was defined as

$$st_{ij}=\arg\left(\max\left(CC_{ij}\right)\right)$$

and the locking as

$$\psi_{ij}=\max\left(CC_{ij}\right)$$

We converted the spike timing differences into phase-values by dividing twice timing difference by the mean spike rate of the respective neurons and then multiplied by π.

$$\theta_{ij}=\pi \left(\frac{2{\mathrm{st}}_{\mathrm{ij}}}{\left(r_{i}+r_{j}\right)/2}\right)$$

### Phase-locking and phase-relation matrix

The matrices represent the phase-locking or the phase-relation between all possible pairs of neurons or oscillators. The diagonal is always 1 (phase-locking with itself) in the phase-locking matrix and 0 in the phase-relation matrix. For the phase-locking matrix the color were from 0 (black) to 1 (yellow-white), if not otherwise stated. For the phase-relation matrix the color were – pi/2 (blue) to pi/2 (red). A negative phase relation (blue) means that the neuron/oscillator X from the x-axis has an earlier/leading phase compared to the neuron/oscillator Y from the y-axis. The phase-relation matrix was threshold for illustration purposes, because phase-relations from non-synchronized neurons/oscillators are randomly distributed over –pi to pi making the plot difficult to interpret visually. The threshold was defined as being equal to 3 times the mean phase-locking value between uncoupled neurons/oscillators.

### Segmentation border analysis

We used the image segmentations performed by several human observers (n = 30) from the Berkeley segmentation dataset (BSDS500,[71]). All subjects did not segment all the images, instead, segmentations from a subset of the observers was available (n∼ = 5) for each image. For each image the segmentation-border analysis based on different observers was averaged. We selected 1-dimensional spatial windows of ±15 pixels centered on segmentation lines that fulfilled the following criteria: (1). A vertical or horizontal segmentation line should consist of three consecutive pixels. (2) Within the spatial window no other line should be present.

For the analysis, the horizontal and vertical line segments were concatenated. We then computed the averaged phase-locking matrix (S4 Fig.) between all oscillators as well as the averaged absolute spatial derivative of contrast values. For computing significance thresholds (permutation testing, [77]) we constructed a null distribution by choosing random positions for the same number of spatial windows.

### Stimulus reconstruction

**Figure 6:**
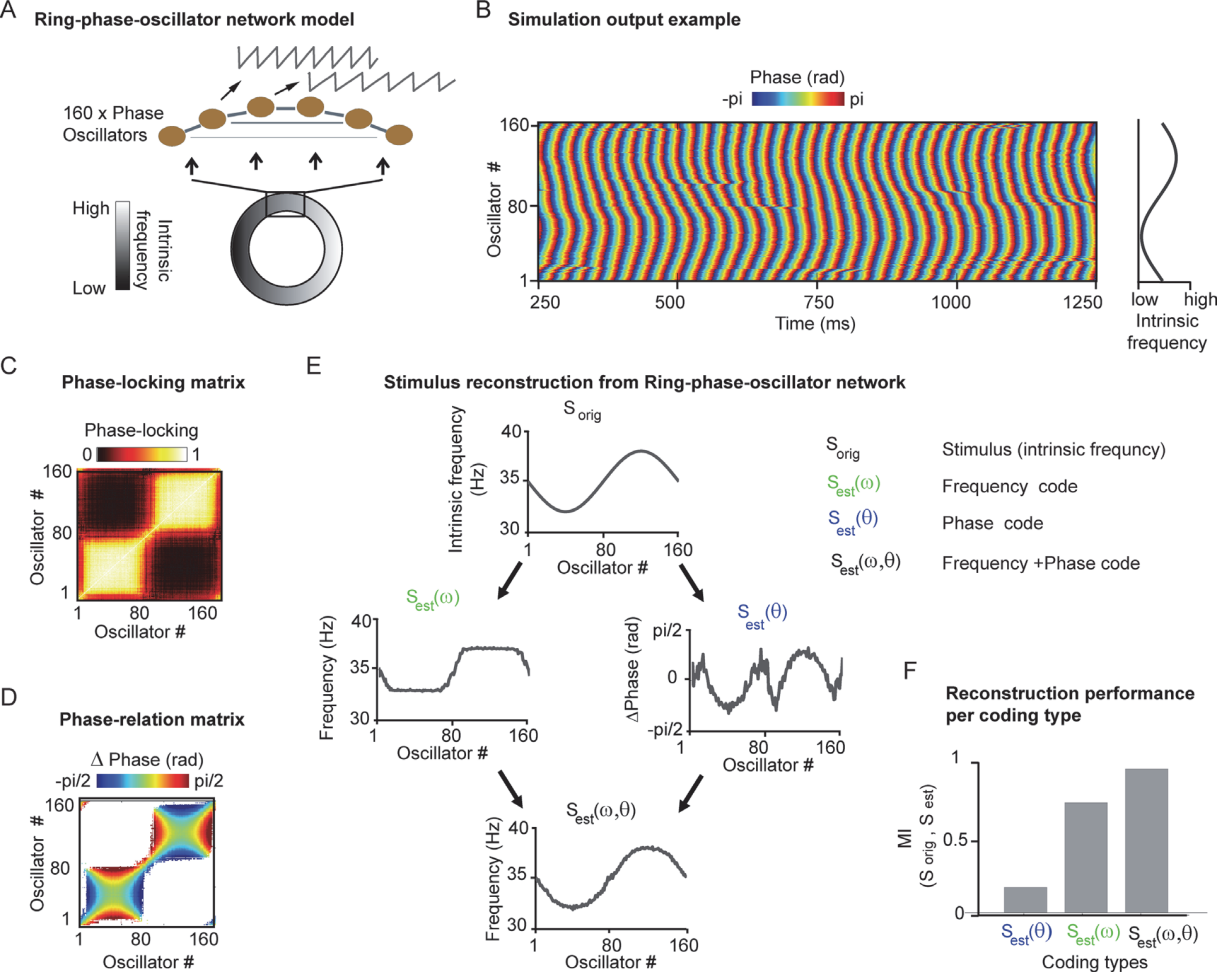
Reproduction of Hodgkin-Huxley results of Figs. 3 and 4 by a phase oscillator model. (A) Replication of Fig. 3 Hodgkin-Huxley network architecture, here with 160 phase-oscillators with the same connectivity structure. B) Example extract of simulation output. Color represents the current phase of individual oscillators. The location of each oscillator in the ring architecture is indicated by the relative input level (intrinsic frequency) C) Matrix of phase-locking values between all possible oscillator pairs, equivalent to Fig. 3F. D) Phase relation between all possible oscillator pairs. Pairs with phase-locking < ∼0.3 (see Method) are masked for illustrative reasons. Blue indicates that the X-axis oscillator leads the Y-axis oscillator, red indicates the reverse. E) Stimulus reconstruction of S_orig_ (intrinsic frequency) based on the frequency code S_est_(ω), the phase code S_est_(θ) and the combined frequency and phase code S_est_(ω,θ) F) The reconstruction performance, measured by mutual information (MI), from lowest to highest MI = 0.17 for phase code S_est_(θ), MI = 0.75 for frequency code S_est_(ω) and MI = 0.95 for combined code S_est_(ω,θ).

The stimulus to be reconstructed S_orig_(i) for each network position i was the excitatory (AMPA) input drive to E-cells for the PING networks and the intrinsic frequency for the phase-oscillator model. For ring-networks the spatial variation of S_orig_ was defined by a sinusoidal function and for the 2D phase-oscillator lattice network by natural image local contrast, where each network position corresponded to one pixel. A seemingly easy way of estimating the stimulus S_orig_ is by using frequency coding *S_est_* (*ω*). If it is defined at single neuron level, it is often termed spike rate. At neuronal population level, the code might be based on the oscillation frequency. In the Discussion section we discuss these different type of frequency coding and their relation (see also S1 Fig.). The spike rate is defined as the number of spikes per second for a given time window (spike count code,[78]). This was used for Fig. 3. The oscillation frequency was determined as the mean instantaneous frequency [79] over the simulation period. This was used for Fig. 2 (LFP), Fig. 6 and Fig. 8 (phase-oscillator) and S1 Fig. (LPA). The *S_est_* (*ω*) was simply defined as the frequency of the neuron i

$$S_{\mathrm{est}}(\omega )=\;\omega_{i}$$

The stimulus S_orig,_ estimated by phase differences between neurons, was defined as follows:

$$S_{est}\left(\theta \right)=(\sum_{j=1}^{j=n}\theta_{ij}\psi_{ij}K_{ij})/n$$

*θ_ij_* is the phase-relation, *ψ_ij_* the phase-locking and *K_ij_* the connectivity strength between the reference neuron i and the neighbor neuron j. *θ_ij_* was determined using the whole simulation period. For analysis based on spike trains, the phase-relation and strength was determined by cross-correlation analysis (see above), whereas for LPA or phase-oscillator analysis it was based on the instantaneous phase variable (for LPA determined by Hilbert transform). The connectivity strength was defined prior to simulation (see above) and was an exponential function decaying over distance (phase-oscillator model) or was of nearest neighbor type with unit strengths (PING network). For all network types, the interaction strength (determined by direct and indirect connections) decayed over distance approximated as an exponential decay function over space with the same parameter used for all network types. Including the connectivity term improved the S_est_, in particular for the 2D phase-oscillator lattice model (MI = 0.60 to 0.67). This is because the detuning-to-phase conversion is coupling dependent. The phase value of each neuron i, was computed by averaging phase-relations to all other neurons in the network weighted by phase-locking strength and coupling strength. *S_est_*(*θ*) is an assembly code using the spike relation between neurons to obtain more information about the stimulus.

For the combined frequency and phase code *S_est_*(*ω,θ*) the stimulus level *S_est_* for a given neuron was then given as

$$S_{est}\left(\omega ,\theta \right)=S_{est}\left(\omega \right)+S_{est}\left(\theta \right)*F$$

where F is a scaling factor determining the contribution of the phase code. The scaling factor F which maximized the stimulus reconstruction performance was chosen. Intuitively, the optimal scaling factor is the slope of the function between intrinsic frequency and the phase variable (see red line in Fig. 2J & 8IV) for a given coupling value. One common scaling factor was chosen for all 80 natural image simulations.

### Measure of stimulus reconstruction performance

We estimated the reconstruction performance as the Shannon mutual information I(X;Y) [80] between the intrinsic frequency image X and the reconstructed intrinsic frequency image Y. The direct method approach gives:

$$I\left(X;Y\right)=\sum_{y\in Y}\sum_{x\in X}p\left(x,y\right)\log\left(\frac{p\left(x,y\right)}{p\left(x\right)p\left(y\right)}\right)$$

where *p*(*x,y*) is the joint probability, *p*(*x*) and *p*(*y*) are the marginal distributions. We normalized *I*(*X*;*Y*) by dividing by *I*(*X*;*X*). We compare the phase-code, frequency-code and the combined frequency/phase code. For the natural images, we computed a baseline reconstruction performance, for each image we computed the normalized MI between the simulation output from that image with the intrinsic frequency maps from all other images. We averaged the 79 MI values (79 MI values per 80 images, each image compared with all other images) to get an estimate of baseline reconstruction for each image. We used a repeated measures ANOVA to test for significant effect of coding types. For post-hoc pairwise comparisons [81] between different coding types we used the Tukey’s HSD (honest significant difference) test (which corrects for multiple comparisons). The Tukey’s HSD was computed as follows:

$$HSD=\frac{\left(\mu_{1}-\mu_{2}\right)}{\sqrt{MSE/N}}$$

With *μ* the mean value of a condition, MSE is the mean sum squared error and N is the number of values within a condition.

## Results

### The frequency of gamma oscillations depends on excitatory input drive

During active information processing, a cortical network will receive variable afferent input drive reflecting sensory variables. By input drive we mean the net excitatory drive to a population of neurons resulting from the sum of afferent excitatory and inhibitory connections. The dependence of gamma oscillations on input drive is central for the understanding of its role in neural processing. Theoretical [17,33,42] and experimental observations [33,42,43,82,83] have shown that excitatory drive increases the frequency of gamma oscillations. For example, recent experimental studies on gamma oscillations in primate visual cortex have shown a striking relationship between visual contrast, which is considered a proxy for excitatory drive [33,42,43], and the frequency and power of gamma oscillations. Results from our own experimental work [33] demonstrated the effect of contrast on gamma oscillations in primary visual cortex V1 and in V2 of rhesus monkeys (Fig. 1A). We found a monotonic increase in the frequency at which the gamma frequency spectrum peaks (Fig. 1B, top) with increased contrast, and a non-monotonic modulation of gamma power (Fig. 1B, bottom).

These findings fit with theoretical studies of the two most common gamma oscillation generating mechanisms [17], the interneuron-gamma network (ING, e.g. [84,85]) and the pyramidal-interneuron gamma network (PING, e.g. [33,86]), which are characterized by increasing oscillation frequency with increasing excitatory drive. We replicated this relationship in a model network consisting of 20 I-cells (fast-spiking type) and 80 E-cells (regular spiking type) using model neurons based on the Hodgkin-Huxley formalism (Fig. 1C, see Methods,[66]). Model neurons interacted through model synapses [23] that included AMPA and GABA-A connections. Each neuronal class received independent external excitatory input, yet the main excitatory input for I-cells was internally generated by E-cells (Fig. 1C). The network exhibited pyramidal-interneuron gamma oscillations (PING) characterized by I-cell spikes lagging E-cell spikes (Fig. 1D). We then (Fig. 1E) systematically modulated external excitatory input to the network (modeled as a train of AMPA-spikes), with the mean level of input ranging from 0.02 to 0.08 milliSiemens per area (mS/cm^2^). GABA-A decay time constant (20ms) was defined such that frequencies were in the range as observed in our own experimental V1 LFP recordings. However, the exact frequency range is not critical for the conclusions of the paper. We observed input-dependent effects on the model power spectra based on the estimated LFP (from transmembrane currents, see Methods). Gamma oscillation frequency increased monotonically with input-drive over a range of ∼20–25Hz [33], as did the spike rates (Fig. 1F, top). Oscillation power (Fig. 1F bottom) showed a nonlinear relationship with oscillation frequency, with peak power at intermediate levels of input [33]. In line with previous work (for review [17]), the main time constant of the PING network oscillation was set by the inhibitory GABA-A decay time constant and by the time needed for the E-cells to escape the inhibition, the latter being reduced by higher excitatory drive. We assume here that synaptic time constants and connectivity strengths did not change within the time-scale considered here for the stimulation (several 100ms to a few seconds).

### Role of detuning and coupling in regulating synchronization and phase relations between two interacting gamma PING networks

As described above, there is substantial evidence that gamma oscillations adapt their frequency as a function of input-drive. But what happens if input-drive varies over cortical space? An experimental study in macaque V1 [43] with contrast-varying stimuli has shown that the frequency of gamma oscillation can vary over a short cortical distance, with higher contrast producing higher frequencies. Hence, nearby cortical location can show different oscillation frequencies. This supports older studies in V1 [31,87] that showed that gamma phase-locking decayed rapidly over cortical distance at the spatial scale of horizontal connectivity. In the light of those findings, a theoretical model of cortical gamma oscillation should be able to express different oscillation frequencies at nearby spatial locations. Such results cannot be simulated in gamma network models that are characterized by global synchronization and express one dominant frequency at a time [88]. Gamma oscillation networks with predominantly local spatial connectivity with locally varying input drive could thus be a promising framework for cortical gamma oscillations. We therefore aimed to gain an understanding of the underlying principles that cause these networks to organize themselves depending on spatially varying input drive.

A theoretical framework for understanding the self-organization principles of such a network with spatially local emerging oscillations is offered by the theory of weakly coupled oscillators (TWCO). The TWCO describes under which conditions interacting (coupled) oscillators synchronize. The ability of a population of coupled oscillators [56] to synchronize is controlled by two opponent forces [55]: their detuning (Δintrinsic frequency) and their interaction strength (here through synaptic interactions), to which we refer as coupling strength. The region in the two-dimensional parameter space of coupling strength and detuning within which synchronization occurs is called the ‘Arnold tongue’ [55]. For conditions within the Arnold tongue (Fig. 2A), the oscillators converge on a common emergent frequency. Within the Arnold tongue, the initial (intrinsic) frequency difference between the pair is replaced by a consistent phase difference, where the oscillator with the higher intrinsic frequency leads in phase. Outside the Arnold tongue, intrinsic frequency differences are maintained, precluding a consistent phase relationship (i.e., phase precession instead of synchronization).

We first illustrate these ideas in simulations from a gamma model consisting of two interconnected PING networks (Fig. 2B, see also [18]). The two PING networks were both identical to the network introduced in Fig. 1C-F, with inhibitory neurons only projecting locally within their own network to excitatory (I→E) and inhibitory cells (I→I). The inter-network connectivity was comprised of excitatory-to-inhibitory connections (E→I, I receiving input from 8 E) and excitatory-to-excitatory connections (E→E, 8 per neuron). In different model simulations, the two inter-network connection types (coupling) were modulated jointly from 0 to 0.07 mS/cm^2^ (note that these values are an order of magnitude lower than in intra-network coupling, see Methods). Fig. 2C shows an example of simulation output with estimated LFP traces (top) and corresponding time-frequency representations (TFRs, bottom). In this example, the drive to the two coupled networks (here coupled with 0.004mS/cm^2^) was very similar (network 1/2 = 0.069/0.0635 mS/cm^2^). This resulted in closely matching oscillation behavior. We then used this model to study the effects of varying input drive differences and of varying coupling strength.

Fig. 2D-F shows the detailed effects of three combinations of coupling strength and detuning (intrinsic frequency difference) on the ability of coupled oscillating networks to synchronize. Phase locking was estimated here based on the population response of each PING network (here LFP, see Methods). In Fig. 2D, network 2 (neurons 101–200 in simulated spike histograms) received (network 1/2 = 0.0598/0.0635 mS/cm^2^) more excitatory input than network 1 (neurons 1–100). Because of a sufficiently small difference in input and intrinsic frequency at the chosen coupling strength (a parameter constellation falling within the Arnold tongue, 0.02 mS/cm^2^), the networks synchronized at a common emergent frequency (∼35.5Hz). This is visible (from left to right) in the overlapping power spectra, in the consistent time difference between spikes of network 1 and 2 in the population spike raster, and in the narrow phase difference distribution (see Methods). The spike raster and the phase difference distribution also show that spikes in network 2 were leading spikes in network 1. In Fig. 2E, the excitation level difference in the networks were approximately reversed (network 1/2 = 0.069/0.0635 mS/cm^2^) while keeping coupling strength constant. Again, the networks synchronized at a common frequency (∼37Hz), but spikes of network 2 now lagged network 1. In Fig. 2F the coupling (cross E-E, E-I) between networks 1 and 2 was decreased from 0.02 mS/cm^2^ to 0.004 mS/cm^2^, while keeping the detuning constant (network 1/2 = 0.069/0.0635 mS/cm^2^), creating a condition falling outside the Arnold tongue region. The two networks therefore did not synchronize but oscillated at different frequencies (network 1/2 = ∼36Hz / ∼38Hz). By systematically modulating the coupling strength and the detuning between the two networks, the Arnold tongue could be fully reconstructed: it emerged as a region of high phase-locking (Fig. 2G) characterized by a common emergent frequency (Fig. 2H) and systematic phase differences (Fig. 2I). Fig. 2J shows a comparison of the intrinsic frequency of one network observed in the absence of coupling (dashed line), and its emergent frequency when coupling was set to 0.04mS/cm^2^ (black solid line). As in Fig. 1F, the intrinsic frequency depended linearly on the input level. However, the emergent frequency during coupling displayed a non-linear function, whereby frequency was constant within the range of the Arnold tongue. Within that range synchronization was observed; meaning that a consistent phase relationship emerged (Δ phase, red line). The phase relationship was linearly related to the input level difference.

### Input-dependent self-organization of a spatially extended gamma network

We described above how the behavior of two interacting PING-networks can be understood in the framework of TWCO. However, to understand the self-organization principles of gamma oscillation activity in a cortical area, one needs to take into account interactions among large numbers of interconnected neurons that constitute multiple potential local PING networks. The local networks may be more easily comparable to anatomically distinct ‘columns’ (which may or may not underlie functionally defined columns) in some sensory systems (e.g., barrel cortex) than in others (e.g., visual cortex), but this correspondence is not critical to our argument. In this study, we used continuous local connectivity and spatially specific input drive as the more general case. We chose a model architecture in which neurons were organized along a ring (Fig. 3A, see Methods), to avoid border effects and thus facilitate analysis. For the generation of the PING mechanism, E-cells and I-cells were designed to have relatively strong local interactions (E-I, I-E) between neighbors (E-I = 0.23 mS/cm^2^, 10 per neuron, I-E = 0.13 mS/cm^2^, 10 per neuron). Inhibitory-to inhibitory connections (I-I) further supported the PING mechanism (I-I = 0.1 mS/cm^2^, 4 per neuron), yet they were not critical [17,18]. In addition, weak but numerous RS to RS excitatory connections (E-E) were added (E-E = 0.03 mS/cm^2^, 25 per neuron) [16]. The topographic input to RS neurons was modulated sinusoidally around the ring (shading in Fig. 3A). I-cells received most of their input drive from nearby located E-cells (for details, see Methods). In the ring-network simulations E-cells had similar spike rates to I-cells and both had spike rates close to the gamma oscillation frequency. This allowed us to use smaller but stably synchronized networks to increase computational efficiency. However, we will describe below that our results can be extended to large sparse-firing gamma oscillation networks in which E-cells (RS) fire much less than the I-cells (FS) and below the gamma frequency.

In Fig. 3B, an example simulation output is shown, displaying the spike raster for the entire network (red, E-cells; blue, I-cells). Neuronal spiking was synchronized in the gamma range (∼25–35Hz), but individual neurons displayed spike timing differences relative to each other that were related to the input-drive differences. Fig. 3C-F describes the detailed relationship between synchronization and input. This relationship will be described both in terms of the strength of phase locking among neurons and of the phase differences among synchronized neurons. Phase locking was estimated by the peak of the cross-correlation histogram computed over the respective simulated spike trains, and the phase difference by the lag of the cross-correlation peak (divided by cycle length, see Methods). We focus here on E-cells but all observations for E-cells have been replicated for I-cells (see similar behavior of I-and E-cells in Fig. 3B).

We found that the spatial extent of phase-locking in the network differed along the sinusoidal input function (Fig. 3C) in close relation to the level of detuning approximated here by the squared input derivative (red line). Neurons receiving their input around the trough and the peak of the spatial sinusoidal input engaged in spatially larger ensembles of synchronized rhythmic activity compared to neurons along the slope of the sinusoidal input (Fig. 3D). To illustrate this, phase locking strength distributions are shown for three reference neurons. Each distribution refers to locking between a reference neuron and all other neurons in the network. The three reference neurons were labeled as neuron ***a*** (red), neuron ***b*** (green), and ***c*** (blue), located respectively near the trough, slope, and near the peak of the sinusoidal input function. The spatial extent of phase-locking (Fig. 3D) decreased with increases in the slope of the sinusoidal input, corresponding to increases in detuning, yielding much larger distributions for neurons ***a*** and ***c*** than for ***b***. Specific features of the input also influenced the distribution of phase locking strength. The spatial distribution of phase-locking for neuron ***b***, situated where the slope of the sinusoid was steepest, was not only small but also symmetric. By contrast, the larger distributions for neurons ***a*** and ***c***, were asymmetric, with a skew towards neurons receiving more similar input drive. Hence, despite the symmetric synaptic spatial coupling for each reference neuron with its neighbors, their spatial phase-locking distributions with neighboring neurons differed. This reflected the spatial variation of input drive to neurons in the vicinity of reference neurons ***a, b*** and ***c***. Moreover, the relation between input drive and synchronization also led to characteristic phase differences among synchronized neurons (Fig. 3E). This is illustrated for each of the same three reference neurons (only phase-relations shown if >.25 phase-locking, see Methods). The reference neurons had systematically leading phase relationships with neurons receiving a lower input drive, and a lagging phase relationship with respect to neurons receiving a higher input drive.

We now consider the combined results of all E-cells in the network. Fig. 3F shows a phase-locking matrix in which the phase-locking values between all possible pairs of E-cells in the network (160 × 160 E-cell pairs) are shown. Neurons around the peak or trough of the sinusoidal input function formed large assemblies of synchronized units. Neurons along the steepest slope of the input function only synchronized with their immediate neighbors (narrow regions of bright color at the centre and extreme ends of the diagonal). Note that neurons close to, but not exactly on, the peak/trough had asymmetric distributions of phase-locking values, in spite of their symmetric connectivity.

In Fig. 3G, phase differences are also shown for all E-cell pair combinations. Within regions of high synchronization, neurons with higher input drive (negative lag shown in blue) led neurons with lower input drive (positive lag shown in red). Both the behavior of phase-locking and phase-relation as a function of detuning are in agreement with TWCO. The detuning magnitude (large at the sinusoidal slope and small around the peak/trough) strongly determined whether neurons could synchronize. If synchronized (within the Arnold tongue), the sign and magnitude of detuning defined the phase-relation.

The synchronization properties of the ring-PING network in Fig. 3 depended on the connectivity patterns. First, sufficiently strong E→I as well as I→E connections were required to allow for PING type synchronization [17]. Further, we observed that the exact synchronization properties depended on the number of excitatory connections in relation to the number of inhibitory connections (S1 Fig.). In the case of more numerous (or stronger) E-E connectivity, the spatial extent of synchronization was larger around the sinusoidal peak compared to the trough. In contrast, in case of strong I-I or I-E connectivity, the spatial extent of synchrony was larger around the trough compared to the peak. This seemingly odd result can be understood if one considers the influence of excitatory vs. inhibitory input in terms of the phase response curve (PRC). Whereas excitatory connectivity will tend to advance the phase of a next spike, inhibitory connectivity will delay the occurrence of a next spike. Neurons synchronizing to other neurons with excitatory connections alone will most optimally entrain neurons with intrinsically lower frequencies (as excitatory connections speed them up) and hence synchronization extends further around the peak. In comparison, inhibitory connections entrain best neurons with intrinsically higher frequencies and will therefore lead to stronger synchronization around the trough of the sinusoidal input. Therefore the balance of inhibitory and excitatory interaction is critical for understanding how PING networks will self-organize depending on input-drive. We stress that future studies should further investigate how connectivity patterns affect the gamma-mediated temporal organization.

In the simulation analysis presented in Fig. 3, network performance was analyzed in terms of simulated spike output, where phase-locking strength and phase differences were derived from spike cross correlations. In experimental studies, gamma oscillation properties are often investigated in terms of the Local Field Potentials (LFP), which is a population aggregate signal (mainly reflecting synaptic potentials, [89]). We therefore conducted a similar analysis on E-cell population activity (representing a LFP-like signal) to test whether the same phase-locking and phase-relation behavior could be observed. Further, we were interested whether input noise affected single neuron spike rates differently than the local population oscillation frequency. To estimate the local LFP-like measure, we aggregated the network spiking activity at each E-cell reference position with an exponentially decaying spatial function (see Methods). We termed this the local population average (LPA), to make clear that it is not the LFP, yet sharing the property of being a population signal. Results are shown in S2 Fig. We observed the same behavior of phase-locking and phase-relation patterns for the LPA estimates. The properties of the network were relatively robust against input noise (S2 Fig.). Generally, the higher the input noise, the smaller the extent of synchrony [18,55]. Further, at higher input noise levels, we observed that the spike rates were no longer highly locked to the local gamma frequency (estimated based on LPA) and, if rates were estimated over a long time window (here 5 sec), the single spike rates could reflect input differences between neurons, despite being locked to the same gamma oscillation frequency. The relationship between population gamma frequency and single neuron spike rates as well as important issues related to noise and the encoding time window will be elaborated further in the Discussion section.

One limitation of the above presented ring-PING network was that the E-cells and I-cells had similar spike rates, both in the range of the local population gamma frequency. However, experimental studies suggest that neurons, in particular pyramidal neurons (RS-type, [66,90,91]), have spikes rates lower (sparser) than the gamma oscillation frequency (they do not spike each gamma cycle). It has been shown in theoretical studies that sparsely firing PING network regimes exist as long as the number of neurons is sufficiently large [92]. We therefore replicated the findings shown in S3 Fig. in a larger network with Izhikevich neuron models [67], which have higher computational efficiency than the Hodgkin-Huxley neuronal model, but still generate realistic RS and FS spiking patterns. The ring-PING network consisted of 4000 E-cells and 1000 I-cells. Whereas the I-cells still spiked close to the gamma range being around ∼40Hz, the E-cells had spike rates around ∼12Hz. Although the E-cells showed spike rates much lower than the gamma oscillation frequency, we still observed the phase-locking and phase-relation among E-cells as described in Fig. 3.

### Detuning is transformed into a complementary gamma phase and frequency code

The results from the two interacting PING-networks (Fig. 2) suggested that reliable phase differences corresponded to small local differences in input (small detuning), whereas frequency differences reflected larger input differences. The same could be observed for the ring-PING network driven by spatially varying input (Fig. 3). Neurons interacting with small detuning (at the peak or trough of the sinusoidal input) exhibited reliable phase differences, whereas neurons interacting at larger detuning values (at the steepest slope of the input function) showed reduced synchrony and large (emergent) frequency differences. This indicates that information about input drive differences might be present both in frequency and phase in a complementary manner.

We therefore extended our analysis of the ring-PING network to investigate neural coding by quantifying explicitly the relationship between the input patterns and the neuronal responses in terms of their frequency and phase-relation. We will first describe the coding types and their derivations. The stimulus (S_orig_) was the spatially-defined sinusoidal excitatory drive to the E-cells. The first coding type was the (emergent) ‘frequency code’ (S_est_ (ω)). We explicitly mean the frequency that would be (experimentally) measurable in a network. In our ring-PING network described above, single neuron spike frequencies (rates) were close to the (LPA) gamma frequency and neurons were strongly locked to the rhythm. Therefore LPA gamma frequencies or single spike rates gave here similar estimates (see Discussion below). The second coding type is the ‘phase code’ (S_est_ (θ)). We described above the phase-relations between neurons in the ring-PING network as function of the spatial sinusoidal input. When neurons were synchronized, hence sharing a common frequency, the neuron with higher drive occupied a leading (earlier) phase. In our network, multiple oscillatory frequencies were present and we therefore had to define the common oscillation frequency by the group of neurons to which it had substantial phase-locking. This was implemented by weighting each phase-relation between neurons by their phase-locking strength. The Arnold tongue relationship states also that a phase-difference between two oscillators depends on their coupling strength. Hence, to achieve more exact estimates of the input differences from the phase differences, we needed to make them independent of coupling. This was implemented by multiplying a given phase-difference by the coupling strength between neurons (see Methods and Discussion). This operation was necessary as a phase-difference between strongly coupled neurons corresponds to a higher input difference than the same phase-difference between more weakly coupled neurons. To summarize, the phase-code was calculated for each neuron as the average phase-relation to all other neurons weighted by their phase-locking strength and coupling strength. In the combined ‘frequency and phase code’ (S_est_ (θ,ω)) both the phase code and the frequency code were summated.

In Fig. 4B, we reconstructed the spatial sinusoidal input (S_ori_) of the ring-PING network based on the E-cell spike count (frequency code S_est_ (ω)), the phase-relation between E-cell spike trains (phase code S_est_ (θ)) or by combining both sources of information (combined code S_est_ (θ,ω)). The frequency code exhibited plateaus around the peak and trough of the sinusoid, where synchronization was strongest. The phase code followed the variation around the peak and trough of the sinusoid, but could not follow the larger input differences (e.g. overall difference between peak and trough). By combing both coding types the variation of the original sinusoidal input was well reconstructed. This was quantified by computing the mutual information (MI, [80], see Methods) between S_orig_ and S_est_. The lowest reconstruction performance was achieved by the phase-code (MI = 0.18), followed by the frequency code (MI = 0.65), with the highest MI for the combined code (MI = 0.92). The contribution of each coding type will depend on the exact input characteristics. For example, the higher the synchrony within a network (e.g. by lower amplitude of the sinusoidal input function) the more information the phase code will add to the frequency code (see discussion). These results indicate 1) that phase coding is most suited to resolve fine (small input differences) and local (high coupling) input variation; 2) that phase coding represents a relative Δrate-phase transform [45]); 3) that phase coding depends on both input difference and coupling strength (Arnold tongue); and 4) that phase coding can add complementary information to frequency coding.

### Extension to a neural phase-oscillator ring-network model

The Hodgkin-Huxley PING network simulations in Fig. 1–4 have shown that the input-dependent gamma synchronization can be well understood within the TWCO and the Arnold tongue [55]. In the following result section, we show that the PING spiking neural network can be successfully reduced to a basic model [52,53] of weakly-coupled oscillator networks, the phase-oscillator model (Kuramoto model, [56]). The reduction to the phase-oscillator model allowed us to investigate the oscillatory properties of much larger topographic networks exposed to natural complex input patterns due to the computational efficiency. However, first we will describe the rationale of reducing local PING networks to abstract phase-oscillators. We will then show that the exact same behavior of the ring-PING network can be reproduced by a ring-phase-oscillator network.

A single phase-oscillator is characterized by an intrinsic (natural) frequency that determines how fast the phase-variable (the central variable of the model) evolves over time. The intrinsic frequency is the frequency that characterizes the oscillator in the absence of interactions with nearby phase-oscillators. A local PING network consisting of a few E- and I- cells is considered here as equivalent to one phase-oscillator (Fig. 5A). The population frequency of the local PING network (LPA, Fig. 5B) would be similar to the frequency of the phase-oscillator. The instantaneous phase [75] of the local population rhythm (Fig. 5C) would be equal to the output-variable of the phase-oscillator model (Fig. 5D). In a network of connected (coupled) oscillators, there is not only a reduction in terms of units (from a number of E- and I- cells to a single phase-oscillator), but also a reduction in the complexity of connectivity. Connectivity (coupling) is defined here in terms of one oscillator advancing or delaying the phase of the other in a manner that is defined by the phase relation between them. Coupling strength refers to the magnitude of the modulation of the phase-variable in two oscillators which is a function of the ongoing phase-relation between the phase-oscillators. This function is referred to as the phase-response curve (PRC). It is a sinusoidal function as defined in the Kuramoto model of weakly coupled oscillators, with a clear attractor at phase 0. For example, if oscillator 1 at a given moment is trailing oscillator 2, oscillator 2 will push the phase of oscillator 1 forward while oscillator 1 will delay the phase of oscillator 2 (assuming sufficiently similar frequencies). This means that given equal intrinsic frequencies, a network initialized with random phases in each oscillator will tend to converge towards the attractor phase. In the case of a local-PING network, the interactions with nearby neurons are exerted through excitatory connections (E-E, E-I) and inhibitory connections (I-E, I-I) which together determine the effective coupling strength and the phase response curve (PRC) of our network. A further difference between oscillator networks and PING networks is the manner in which input modulates ongoing interactions in the network. In PING networks, the oscillation frequency emerged from the interaction between network properties and excitatory input drive. In phase oscillator networks, the intrinsic frequency of a phase-oscillator was set before simulation based on a function (as established experimentally) linking input strength to oscillation frequency. Further, whereas in the PING network the gamma rhythm might be not sustainable in some conditions, e.g. due to low input drive, a phase-oscillator will always oscillate at any arbitrary frequency. Overall, it must be emphasized that even though not all complexities of the PING network can be captured by a phase-oscillator model; we argue that it captures the most characteristic properties of PING network behavior.

To illustrate that phase-oscillator networks capture the behavior of the PING model, Fig. 6 describes a ring-phase oscillator neural network similar to the ring PING network (Fig. 6A), in which 160 phase-oscillators were locally coupled along a ring (see Methods). The intrinsic frequency of each phase-oscillator was set by a sinusoidal input function (defined over the ring). The simulation output can be seen in Fig. 6B, which shows the output phase-traces of the 160 phase-oscillators. In Fig. 6C-D, we computed the phase-locking and the phase-relation matrix between all phase-oscillators. The results shown resembled those obtained for the ring-PING network (Fig. 3F-G). The spatial extent of phase-locking was larger around the peak and trough of sinusoidal function. The phase-relation patterns within the region of phase locking were the same as in the ring-PING-network, so that phase-oscillators with higher intrinsic frequency had a leading phase compared to other phase-oscillators. In Fig.6 E-F, we tested the contribution of the different coding types to input reconstruction (see Fig. 4B-C for comparison). Notice that S_orig_ (stimulus input) corresponds here to the intrinsic frequency set by an input function and not to excitatory drive. The same results (here with sinusoidal intrinsic frequency fluctuation of +/−3Hz) were obtained as in the ring-PING network. The best input reconstruction was given by the combined frequency and phase code (MI = 0.95), followed by the frequency code (MI = 0.73), and lastly the phase-code (MI = 0.17). We also reproduced the asymmetries in spatial synchronization (around peak and trough of sinusoid) which in the ring-PING network were induced by changing the amount of excitatory connections in relation to the amount of inhibitory connections (see S1 Fig.). The asymmetries in spatial extent of synchronization were obtained in the phase oscillator network by modifying the phase-response curve (PRC). To model a dominance of excitatory connections, we set all values below zero (phase delay) to zero, and to model a dominance of inhibitory connections we did the opposite. This resulted in the same asymmetries in synchronization around the peak or trough of the sinusoidal function (S2D Fig.).

### Large 2D topographic phase-oscillator networks driven by natural spatial input-patterns self-organizes along the same Arnold tongue principles with implications for coding and spatial synchronization

**Figure 7:**
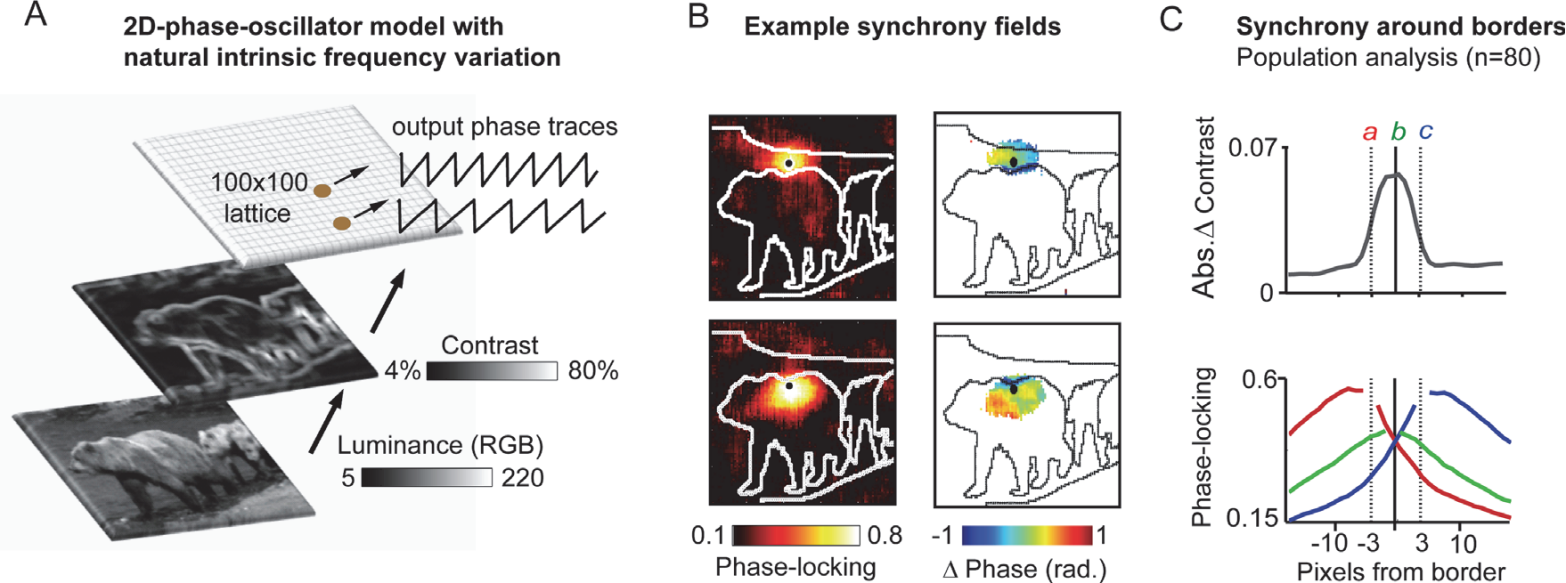
Phase-oscillator model with natural image input. A) The general approach: The natural image was compressed to 100×100 pixels and transformed from a luminance image into a contrast image. Contrast values were used to define the intrinsic gamma-frequencies of the 100×100 phase-oscillator lattice on a one-to-one pixel to oscillator basis. B) Example synchrony fields (color) of two reference oscillators to all other oscillators (color) overlaid onto border segmentation of the corresponding image. One example (black dot) was located outside of the main object (top row) and the other within the object (bottom row). Phase relation maps of example oscillators are shown to the left that represent the phase relation to all oscillators with phase-locking >0.3 (see Methods). Blue (red) indicates that the oscillator leads (lags) compared to the reference oscillator. C) Segmentation-border triggered analysis. From the online image database [71] segmentation borders as indicated by 30 human subjects are available. We used these segmentation borders to analyze spatial synchronization around them (see Methods). Borders are thought to be associated with high contrast variation (and hence detuning) [112]. Top plot shows the mean absolute contrast spatial derivative (averaged over the 80 images) confirming that segmentation borders are indeed associated with higher contrast/detuning. Below the mean synchronization profile for reference oscillators a and c located 3 pixels on each side of the window’s center (i.e., the boundary location) and reference oscillator b located at the boundary location. Spatial synchronization is reduced over the segmentation border in line with the higher detuning at the segmentation borders (see S4 Fig. for more details).

We took advantage of the computational efficiency of the phase-oscillator model and extended our analysis to a 2D 100×100 lattice networks. The network consisted of 10.000 phase-oscillators with a total of 10^8^ possible connections. Connections among phase-oscillators decreased exponentially in strength as a function of distance on the lattice as an approximation for cortical horizontal connectivity (e.g. V1, [73]). Note that the lattice network was an abstract model of a cortical area aimed to reflect only the essential characteristics of a sensory cortical area (topographical map, spatially local connectivity, feature map). The model was aimed to capture the essential input-dependent self-organization principles of cortical oscillations, in particular gamma oscillations. We tested the network behavior using visual images representing natural and complex intrinsic frequency variation better (Fig. 7A). Inspired by experimental observations of a close link between visual contrast and gamma oscillation frequency in macaque visual cortex V1 and V2 [33], we used the local contrast of natural visual stimuli to define the intrinsic frequencies of the phase-oscillators. To that aim, we used 80 (grayscale) natural images from an online database (Berkeley segmentation dataset, see Methods). The images were down-sampled such that each pixel of the image corresponded to one phase-oscillator. Local contrast was estimated using a root-mean square measure [25] with a spatial kernel of 3 pixels. From the online database, information on the location of boarders between objects, or segments in the image (segmentation borders), defined by 30 human observers [71], was also available.

In Fig. 7B, we show in the left column the synchronization (black = no phase-locking, yellow/white = max phase-locking of 1) and in the right column the phase-relations (blue = earlier phase, red = later phase, white = below threshold, see Methods) of two reference oscillators (black dots) as compared to all other oscillators in response to an example image. We will use these examples to show how the synchronization fields and phase-relations adapt to local changes of contrast/intrinsic frequency. The ‘synchronization field’ refers to the spatial extent over which a reference oscillator synchronizes with neighboring oscillators [46]. The synchronization fields were asymmetric, despite symmetric coupling in the network. Within the synchronization fields, off-zero phase-relations could be observed. This was due to the positions of the two example reference phase-oscillators, which were chosen to be close to an object border (here a bear) with one reference oscillator located just outside the main object (top row Fig. 7B) and the other just within (bottom row Fig. 7B). The object border was associated with high local contrast variation (hence large detuning). The asymmetry of the synchronization fields followed from the fact that synchronization drops with the rapid increase of local contrast when moving from the reference oscillator towards the border (increasing detuning) while the converse was true when moving from the reference oscillator away from the border (decreasing detuning). Here, synchronization extended far from the reference oscillator towards the interior or ulterior surfaces (because the small detuning within surfaces permits synchronization over larger spatial extents). Furthermore, within the synchronization fields, the oscillators closer to the border led in phase compared to the reference oscillator, because the border had higher contrast/intrinsic frequency, while the converse was true when considering oscillators away from the border. These example synchrony fields indicate that phase-oscillators in the 2D lattice model behaved similarly to those in the ring-phase oscillator and ring-PING network models. Moreover, the data suggest that the synchronization fields might capture specific aspects of the statistics of contrast distributions in natural images. To further explore this point, we tested systematically in Fig. 7C how synchronization was affected around segmentation borders in 80 natural images (available from the Berkeley segmentation dataset [71], see Methods for more details). We first tested whether contrast variation was significantly modulated around segmentation borders (see Methods). To quantify these effects, we defined 1-dimensional spatial windows of ± 15 pixels centered on segmentation borders. We then aligned the different windows and averaged them for each image separately (see Methods for more details). Population statistics are based on these average windows per image. We then calculated the averaged absolute spatial derivative of contrast values (equivalent to detuning) along each window. Fig. 7C (top) shows a steep change in contrast as a function of distance to the border. Mean contrast variability at the center of the window, at the border, was significantly different from the extremities of the window (paired t-test: t = 7.35, df = 79, p <0.001). We then quantified the change in synchronization as a function of distance to a border. For initial population analysis (N = 80), we selected three reference oscillators. Reference oscillators ***a*** and ***c*** (Fig. 7C bottom) were located 3 pixels away from each side of the border, and reference oscillator ***b*** was on the border. Relative to reference oscillators ***a*** and ***c***, synchronization fields showed a much more rapid decline of phase-locking strength towards the border than away from the border (see S4 Fig. for statistics and more details). Thus overall, the asymmetry of synchronization fields around reference oscillators ***a*** and ***c*** in Fig. 7E matched with the asymmetry of synchronization fields around neurons ***a*** and ***c*** in Fig. 3C-E of the ring-PING network model. This input-dependency of spatial synchronization suggests, in line with previous studies [21,29,46,47,93,94], that oscillatory synchronization might be a useful tool for clustering operations, for example for visual segmentation.

**Figure 8:**
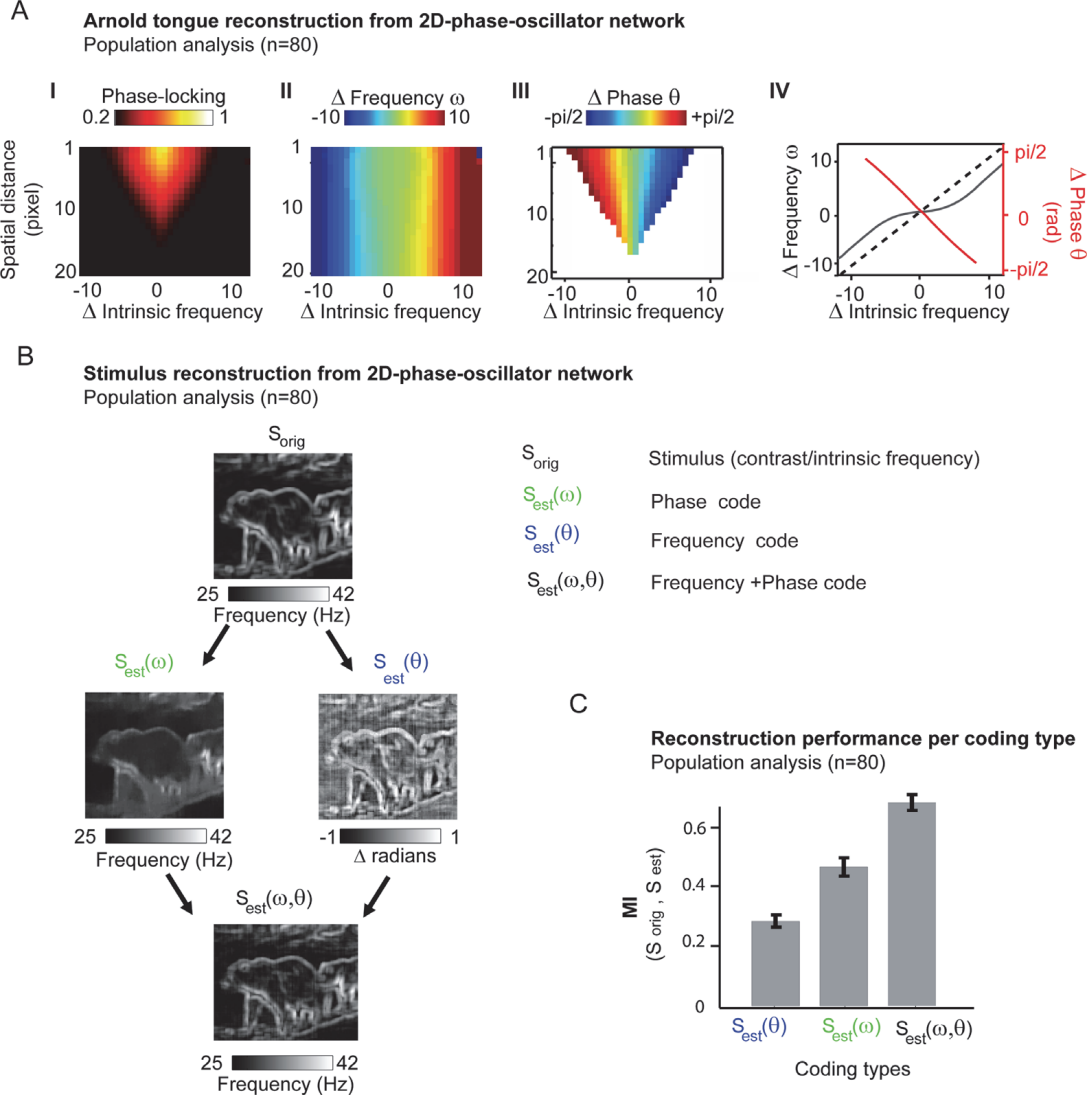
Arnold reconstruction and information content of the frequency, phase and combined codes for natural image input. A) Arnold tongue reconstruction from the natural images (n = 80) by using detuning and coupling between all possible pairs of oscillators in the lattice network. We used lattice distance here as approximation for coupling (see Methods). The resulting Arnold tongue in terms of (I) phase-locking (II) phase-relation and (III) (emergent) frequency difference is shown. In (IV) a cross-section of the Arnold-tongue with overlaid representation of phase-locking (black), phase-relation (red) and intrinsic frequency (dashed) for an oscillator-pair of a lattice distance of 3 pixels (direct coupling strength = 0.62). B) Stimulus reconstruction of the natural image contrast. Intrinsic frequency of all phase oscillators determined by local contrasts of example image S_orig_ [71] were reconstructed based on the frequency code S_est_(ω), the phase code S_est_(θ) and the combined frequency-phase code S_est_(ω,θ) C) The reconstruction performance, measured by mutual information (MI), was from lowest to highest MI = 0.28 for S_est_(θ), MI = 0.46 for frequency code S_est_(ω) and MI = 0.67 for combined code S_est_(ω,θ). Error bars give ±3 SEM (n = 80).

So far, we have described the behavior of the phase-oscillator model using particular image examples. We now analyze the network behavior in terms of the principles that underlie its self-organization behavior independent of the specific image providing the input. If the input-dependent self-organization of the phase-oscillators is mainly governed by principles of the TWCO, then one should be able to reconstruct the Arnold tongue from the simulation output. To test this, we determined for each phase-oscillator pair its coupling strength (direct connections) and detuning (as derived from the 80 input images). Fig. 8A shows that in the two-dimensional parameter space of detuning and coupling strength an Arnold tongue could be observed in terms of phase-locking (Fig. 8A I), frequency difference (Fig. 8A II) and phase-difference (Fig. 8A III). Fig. 8A IV represents a horizontal cross-section of the Arnold tongue (coupling strength = 0.62) where frequency differences (black) and reliable phase differences (red) are plotted. The dashed line denotes the intrinsic frequencies. As described in Fig. 2 for PING networks, phase differences better resolved smaller intrinsic frequency variation, whereas frequency differences reflected best the larger differences. The Arnold tongue reconstruction was reliable and reconstructions from individual images looked very similar to the averaged one shown in Fig. 8A. The Arnold tongue properties were similar to the one described from the two interacting PING networks (Fig. 2G-J). This analysis confirmed that the phase-oscillator lattice model with complex natural detuning (intrinsic frequency variation) behaved very similar to the ring-PING network model driven by simple sinusoidal excitatory drive. Hence, information about the natural image stimulus should be available in a complementary manner at the level of frequency variation as well as phase variation. We therefore quantified the amount of information present in the above defined coding types, frequency and phase coding, as well as a combined coding type (Fig. 8). We used the same approach as used for the ring-PING network and ring- phase oscillator network. The stimulus S_orig_ to be reconstructed was the intrinsic frequency image defined by the local natural image contrast. The frequency code S_est_(ω) was the mean (emergent) frequency of a phase-oscillator. The phase code S_est_(θ) for a given phase-oscillator was the phase differences with all other phase-oscillators weighted by phase-locking and coupling strength (see Methods). The combined code S_est_(ω,θ) was the summation of both former coding types. In Fig. 8B, the stimulus and the reconstruction estimates are shown for an example image. The stimulus reconstruction based on frequencies S_est_(ω) appeared smoothed compared to the original stimulus S_orig_ reflecting the loss of fine spatial details. The reconstruction based on phase-relations S_est_(θ) resembled a second derivative of the original stimulus S_orig_. The phase code reflected well local and fine details but it did not reflect the absolute contrast/intrinsic frequency level. A fair reconstruction of the original stimulus was achieved by the combined frequency and phase code S_est_(ω,θ), which indicates that information from the frequency code and phase code were complementary. In Fig. 8C, we quantified information content, by estimating the (normalized) mutual information (MI) between the intrinsic frequency image S_orig_ and the reconstructed image estimates S_est_ ([80], see Methods) For the example image in Fig. 8B, the MI was 0.44 for S_est_(ω), the MI for S_est_(θ) was 0.31, and the MI for S_est_(ω,θ) was 0.69. Over the population of 80 natural images (Fig. 8c), the MI was 0.46 (SEM = ±9*10^−3^) for the frequency code S_est_(ω), 0.28 (SEM = ±5 *10^−3^) for the phase code S_est_(θ) and 0.67 (SEM = ±7*10^−3^) for the combined frequency and phase code S_est_(ω,θ). A repeated measures ANOVA showed that all three codes were significantly different from one another (F(2,158) = 881, p<0.001), and all pair-wise comparisons were highly significant according to the Tukey’s HSD tests).

## Discussion

In the following, we will first discuss the underlying assumptions and limitations of our cortical gamma network model and relate them to previous modeling approaches. Then, we will turn to the implications of our findings for gamma phase coding and its relation to frequency/rate coding, stressing the distinction between the coding of larger versus smaller input variations, and the effect of noise, encoding time window and connectivity. Further, we will discuss the experimental and theoretical implications of the relative rate-to-phase transform, as proposed here for gamma synchronization, compared to an absolute rate-to-phase transform. This is followed by considerations on input-dependent spatial synchronization, in particular in the context of the phase-oscillator network response to natural images, and comparisons to related modeling approaches more specifically designed for image segmentation. We end with testable experimental predictions that follow directly from the present study.

### Underlying model of cortical gamma oscillations

**‘Single oscillator model’ vs. ‘multiple oscillator model’**. The exact underlying mechanism of gamma oscillations is still under debate, even though significant advances have been achieved over the last decades. A primary distinction [17] has been made between interneuron-network gamma (ING) versus pyramidal-interneuron network gamma (PING). However, we will first focus on another key model distinction that has received much less attention so far. In one class of models, inhibition acts as reference clock to which excitatory neurons at different cortical locations are entrained at different phases [15,84,95,96], whereas in another class of models I- and E-cells at different cortical locations represent different ‘clocks’ (oscillators) that synchronize at different phases [18,52,54]. We call the former the ‘single-oscillator’ model and the latter the ‘multiple-oscillator model’. The critical distinction is whether I-cells receive spatially local excitatory drive or whether the I-cell network acts as a (indiscriminative) single unit. In the latter case, the network can express only one dominant gamma frequency at a time. In principle, both types of models can be implemented in either PING or ING mode. Reliable phase-coding in a single oscillator model regime is not easy to achieve [18] and requires strong inhibition and relatively high spike timing precision. Phase-coding is determined mainly by how fast E-cells recover from inhibition (similar to latency coding, [97]). In the ‘multiple-oscillator model’ a precise entrainment of E-cells by their nearby I-cells is not essential as long as a gamma rhythm is produced. Phase-relations are established between nearby gamma rhythmic E- and I-cells through synchronization [55], during which phase-relations are determined by detuning and coupling strength. In our network models, we observed that the behavior of the PING network was largely consistent with the multiple-oscillator model. However, the two models do not necessarily exclude each other.

**ING versus PING**. It has been shown that an ING [83,84] network of mutually interacting inhibitory neurons can produce robust gamma oscillations, if the network is driven with sufficient excitatory input. In this model, local pyramidal neurons receive rhythmic inhibition from the I-cells. An extension of the model is the PING model, where the excitatory drive to the I-cells originates from the local pyramidal E-cells themselves [15,86]. In the PING model, the excitatory state of the pyramidal neurons influences the network rhythm, in particular the frequency. In comparison, in the ING model the frequency is determined solely by the excitability of the inhibitory neurons.

Both ING and PING mechanism likely coexist in cortical networks [92] and the dominance of one to the other may switch depending on network state [90]. Experimental studies suggest that cortical gamma oscillation in a stimulus-driven state show properties consistent with the PING model [15,90]. However, the essential TWCO properties we described in our networks are not restricted to PING networks. An ING network with locally defined connectivity and excitatory drive will exhibit similar behavior. Hence, our modeling results are expected to be independent of specific PING and ING network configurations. However, in the case of E-cells receiving rhythmic inhibition from an ING network that either does not receive a spatially-defined drive or is coupled in a manner such that the ING network acts as a single unit, then the regime is not expected to be in agreement with TWCO (‘single-oscillator model’, see above). The same is expected for a PING network where the E→I connections are all-to-all, such that all I-cells receive the same mean input from E-cells.

**Assumptions of the theory of weakly coupled oscillators**. Hoppenstaedt and Izhikevich [52] formulated two basic assumptions in which oscillatory interactions in the cortex would be expected to occur in the regime of weakly coupled oscillators. First, they assumed that oscillations are internally (autonomously) generated and second, they assumed that the coupling is weak between oscillating neural populations.

The first assumption was fulfilled for our study by the PING mechanism. The mean network input had no rhythmic components. Oscillations were generated by the interaction between E-and I-cells. The second assumption of weak coupling can be understood as oscillatory interactions between units leading to phase shifts (as defined by the PRC), but not to substantial amplitude changes or perturbation of the rhythm-generating mechanism (or quenching, [55]). In the case of the two PING networks (Fig. 2), the cross-network connections were an order of magnitude smaller than the within-network connections. We observed small phase-dependent amplitude fluctuations (corresponding to a partially synchronized state), but they did not substantially affect the phase-trajectory of the network oscillations. In the ring-PING network (Fig. 3), connectivity was spatially continuous and no columnar structures were assumed, thus there was no distinction between within and cross network connectivity. The synaptic connectivity strengths were in the range as normally used for PING networks [18] and the behavior was stable for a large range of different connection strengths. As described in Fig. 5, for the reduction of the ring-PING network to the ring-phase-oscillator model we assumed that single phase-oscillators could be equated to pairs of E-cells and I-cells of the PING network. Further, we assumed that the complex interactions (E-E, I-E, E-I, I-I) could be approximated by a single PRC-defined connection type. Weak coupling in this context means that the synaptic coupling between neurons did shift the spike timing and but neither increased the firing rate substantially nor interfered with the spike generation mechanism. The comparison of the ring-PING network with the ring-phase oscillator network indeed revealed striking similarities (compare Fig. 3 with 6). In conclusion, we showed that our weakly coupled oscillator network conformed to the assumptions of the TWCO. In addition, our modeling data indicates that discrete columnar network structure (coupling of many individual PING networks) is not a necessary condition to investigate PING-type oscillations in the weakly coupled oscillator regime.

Note that principally, the TWCO can be applied to any frequency band. However, to apply the TWCO in a valid manner to rhythms recorded in the brain, the mechanism generating the rhythm should be characterized by a link between excitation and intrinsic frequency, and by the possibility for different frequencies to exist in neighboring neural populations. Arguably, the generative mechanisms are best understood for gamma [17], and the emergence of localized differences in frequency has to the best of our knowledge only been demonstrated in the gamma range [43]. Therefore, the implications of our work are meant to be restricted to gamma oscillations.

**Networks with sparsely firing neurons**. In Fig. 3, the E-cells had firing rates close to the gamma oscillation frequency (∼30–40Hz), which might be considered as unusual for regular spiking pyramidal neurons (RS, [66]). E-cells often spike at much lower rates than the gamma frequency, and in the hippocampus spike rates can be as low as a few Hertz despite gamma oscillations in the 20 to 80Hz range [98]. It has also been described in the neocortex that Layer 2/3 networks display more sparse-firing properties compared to Layer 4 [99]. In contrast, I-cells of the fast-spiking type (FS) have firing rates that can be close to the network gamma rhythm [15]. To demonstrate that the network behavior described above was not restricted to networks with fast firing E-cells, we constructed a network in which the E-cells had much lower spike rates. It has been described that such ‘sparse’ networks need a sufficient number of neurons to reach stability [92], as each cell contributes a spike only every few cycles. For computational efficiency we used Izhikevich-type neurons [67] instead of Hodgkin-Huxley (HH) type to increase the network size by an order of magnitude. We were able to replicate the findings from the smaller HH-network in a large Izhikevich-type network where E-cells had 3–4 times lower firing rates than the gamma oscillation frequency. Hence, the essential behavior described generalizes to more sparsely firing networks.

**Topographic phase-oscillator lattice model**. The topographic phase-oscillator neural network was aimed to represent a simplified sensory cortical area (inspired by V1) [73] with predominantly local connectivity where input features are smoothly represented over cortical space (feature map). We emphasize however that our topographic network model is not a strict model of a particular sensory area (such as V1 with columns and hyper-columns, layers). Moreover, we used natural visual contrast stimuli as an example for natural input pattern to a visual topographic network, but the described principles of self organization may well apply across different sensory systems. However, we suggest that the main principles underlying the network self-organization are perhaps most easily experimentally testable in a cortical area like V1.

**Conduction delay and asymmetric connectivity**. In the PING networks the conduction delay in the interaction was mainly determined by the synaptic postsynaptic potentials [1–2ms]. The conduction delay did not change with distance between neurons. However, conductional delays of horizontal interactions over cortical space can be significant [73,74]. Conduction delays may affect the phase-relation as well as phase-locking. For the phase-oscillator lattice model we included a time-delay term as a linear function of spatial distance (slope of 0.4 ms/pixel, offset 1ms). By including this term we observed that the spread of spatial synchronization became more limited, helping to restrict the ‘synchronization fields’. We did not include the time-delay term explicitly in the reconstruction coding formula, but because it affected the phase-locking strength, it was implicitly included in the weighting of phase-relations by phase-locking.

An additional factor that is of importance in our models was the assumption of symmetric coupling, and how it interacts with time-delays. Whereas under conditions of symmetric coupling we expect time-delays to affect phase-locking but not phase-relations, under asymmetric coupling we expect pronounced effects also on phase-relations [59]. More research will be needed to investigate the effect of time delays and asymmetries in connectivity on input-dependent gamma synchronization.

### Implications for neural coding

**Background.** The problem of neural coding of sensory signals is a central topic in neuroscience with a long history (for review [1,100–102]). Despite substantial advances over the last decades, many fundamental issues remain unresolved. We will give a short review of different perspectives on this issue. A first important conceptual opposition in the literature is that between rate and time coding. The ‘rate-coding hypothesis’ has a long tradition founded on early discoveries of a close relationship between spike rate (frequency) and sensory variables. Rate-codes however are constrained by the length of the encoding time window (integration time constant), which defines its resolution. Another limitation is represented by the saturation properties of many spiking neurons in the low and high input range, that is, the limited dynamic range of neuronal spike rate. In this framework, variability in spike times over time is considered as noise to be averaged out either over time, or over many neurons. As an alternative to rate coding, the ‘time-coding hypothesis’ was introduced later, stating that precise spike timing contains a significant amount of information about the stimulus [103,104]. Time-codes require short integration time windows (coincidence detectors, [105]). It is well established that single-neuron spike timing contains additional information about time-varying stimuli [102]. However, the idea that spike-timing relation between different neurons is meaningful has received less acceptance in the neuroscience community [2]. A further distinction has been made between ‘independent single-neuron coding’ and ‘assembly/ensemble coding’. In an ‘independent’ scenario, each neuron codes its input by its rate and position within a feature map (position coding) independent of other neurons. All the information is represented in the rate of the single neuron (input-rate transform) and the position it has within the network. However, experimental observations have shown in many studies [106] that correlations exists between spike trains at various spatial and temporal scales. We consider here precise correlations of spiking timing between neurons (synchrony) in contrast to slow time varying (trial-by-trial) fluctuations in spike rate (known as noise correlation, [107]). There have been various experimental studies showing precise spike synchrony between neurons on a fast-time scale [21,108,109]. The observations of spike synchrony indicates that additional information about the stimulus might be present in the relative spike-timing between neurons, which has led to the formulation of the ‘assembly/ensemble coding’ hypothesis [3,6,47,110]. Here, information is represented in the exact spiking pattern of several neurons. The relationship between oscillation phase coding (assembly code) and single spike rates or population frequency has not been well studied.

In the following sections, we will discuss in more depth the implications of our theoretical results for the understanding of gamma phase coding and its relationship to rate/frequency coding.

**Complementary coding between frequency and phase**. Our theoretical analysis has shown that oscillating neural networks with local connectivity represent input patterns in frequency and phase according to the TWCO. Of particular importance is the Arnold tongue that describes, as a function of coupling strength and input difference, the transition of frequency coding to phase coding. Reliable phase coding can only exist if neurons are synchronized (phase-locked, [18]), or in other words converge on a common frequency. That is the reason why frequency and phase coding are in principle complementary, because the process of synchronization ‘transfers’ the information (detuning magnitude) represented at the level of frequency differences into phase differences (outside vs. inside the Arnold tongue).

We have shown in our models that phase coding can add significant information about the stimulus. The level of contribution of phase coding will depend both on the input as well as on the coupling characteristics. A network will rely more on frequency coding if the coupling (interaction) between neurons is weak/sparse and input variability is high, whereas it will rely more on phase coding if coupling is strong and input variability is low. We argue that in many cases the network will be situated between these two extremes and would profit from combining both coding types.

Previous theoretical studies have already suggested the TWCO as the underlying model of phase-coding. Hoppenstaedt and Izhikevich [52] discussed the theory of weakly coupled oscillators in the context sensory cortical columnar processing. Moreover, Tiesinga and Sejnowski [18] discussed the behavior of multiple interconnected PING networks using TWCO. They were able to reproduce the experimentally observed gamma phase coding of stimulus orientation in primary visual cortex [34]. In their modeling study, each PING network had a different orientation tuning. During ‘presentation’ of a particular stimulus orientation, the PING networks synchronized and the PING network with strongest input (optimal orientation) led in phase. In other words, the PING networks operated within the Arnold tongue regime (in which detuning was translated into phase differences). They also described that PING networks with weak coupling showed stronger phase shifts than PING networks with stronger coupling, in line with experimental data [34]. We replicated the main observations of Tiesinga and Sejnowski [18], but also extended their results. First, we confirmed their observations also in networks with continuous spatial connectivity (no assumption of columns). Second, we described in a systematic way networks which ‘translated’ input/intrinsic frequencies into phase-relations as well as (emergent) frequencies. Moreover, we quantified explicitly the contribution of frequency and phase information to the encoding of simple as well as complex natural stimuli.

We described above the fundamental complementary nature of frequency and phase coding, as predicted by the Arnold tongue. However, in assessing the complementary aspects of frequency and phase coding, several factors need to be taken into account. First, the transition between frequency (asynchrony) and phase coding (synchrony) is not sharp, but characterized by a state of partial synchrony in which both frequency and phase coding can be expressed. Second, noise has an important effect on the level of synchrony/partial synchrony. Third, the encoding time window is critical for the assessment of the contribution of phase and frequency coding. These points are also critical to evaluate the relation of oscillation frequency to single spike rates as described below.

**Noise and partial synchrony**. Our study indicates that oscillators with different input strengths/intrinsic frequencies are not necessarily precluded from synchronization, however, in order to synchronize they must first arrive at an emergent ‘compromise’ frequency. This is apparently at odds with experimental studies showing that different neurons can be phase-locked to a rhythm, yet still express different spike rates [34]. Further, this seems at odds with a study that reported significant gamma synchronization between separate neuronal populations despite different (mean) gamma frequencies [43]. Importantly, their findings can be accounted for within the TWCO by ‘partial synchrony’ [55], corresponding to an attraction towards a synchronous state during brief time periods, including the adoption of a common frequency, interspersed with periods of asynchrony and separate frequencies.

Noise, largely present in biological systems, plays an important role. As reported also by Tiesinga & Sejnowski [18], noise shrinks the border of the Arnold tongue, and increases the amount of partial synchrony [55]. Arnold tongue reconstruction from the PING-networks (Fig. 2) and the phase-oscillator lattice model (Fig. 8) exhibited large regions of partial synchrony with only a very small region where phase-locking was perfect (close to 1). Therefore, because partial synchrony inter-mixes states of synchrony and desynchrony (inside/outside of Arnold tongue) information about stimulus input in a noisy network will be represented (on average, yet not at the same time) in both the frequency as well as in the phase differences.

**Single spike rates and encoding time window**. Single neuron spike rates will be close or equal the common population frequency if neurons are highly synchronized to the population rhythm (as in Fig. 3). In noisy and sparsely firing networks however, the synchronization can be low and a single neuron spike rate can be relatively independent of the population frequency (yet still exhibit phase coding, [18]). This applies particularly to neurons with spike rates much lower than the gamma oscillation frequency, because higher-order Arnold tongues are narrow and hence much more sensitive to noise. This fits with experimental data where a rather low locking of single neurons to an oscillation rhythm is observed, especially for pyramidal neurons [90]. In this regime, single neuron spike rates contain additional information compared to the local population rhythm (see S2 Fig.) and both phase-relation and single neuron spike rate might contain overlapping (‘redundant’) information.

Yet, an important aspect to consider, is how much time is needed to reliably retrieve the relevant information [78,103,104]. Compared to a rate code, the phase-code can retrieve the information in much shorter time windows (in one or a few oscillation cycles). For example, considering a rate code, a 1Hz spike rate difference can be retrieved with a minimal 1000ms encoding (integration) time window, whereas with the phase-code a time window of 25–100ms might be sufficient for a 40Hz oscillation (1–4cycles) to get reliable estimates. Hence, although information about small input differences might be present in both phase-relation and spike rates of single neurons over longer time windows, a phase-code could be particularly beneficial for stimulus reconstruction on a fast-time scale (e.g. within typical saccade intervals of ∼300ms).

**Δrate-phase transform**. Analogous to the common input-rate transform of single neurons, it has been assumed that oscillations implement a rate-phase transform [15,44,45]. That means that the higher the input strength to a neuron, the earlier in phase the neuron will spike within the oscillation cycle. Therefore the phase gives a direct estimate of the absolute input levels. There is experimental evidence for this type of transform for slower frequency oscillations, in particular the delta/theta rhythm [44,45]. Strong experimental evidence has been obtained from hippocampal theta oscillations, where neurons spike at different phases depending on their input [111]. An explicit experimental test for a rate-phase transform was carried out by McLelland&Paulsen [45] for the hippocampal theta oscillations. They found that whereas theta oscillations implemented a rate-phase transform, they did not observe the same for gamma oscillations. This seemed to be in line with other observations that did not find a systematic relation between the input level and the gamma phase under natural stimulation conditions [44]. In addition, stimulus contrast modulations did not yield phase shifts in macaque V1 [43]. However, other experimental studies in macaque V1 have shown that gamma phase can code for orientation tuning [34], suggesting a role of gamma phase in stimulus encoding. Hence there are conflicting results whether gamma phase coding can represent input in the same way as has been reported for slower oscillations. Our computational analysis might help resolve these seemingly contradictory results. We have shown, in line with previous studies [18,52,54,59], that the gamma phase coding can be understood in terms of the Arnold tongue, where phase-relations depends on the input difference/detuning (for a given coupling value). Hence, the essential parameter is not the absolute input level, but the input difference between interacting neurons. We term this coding the ‘Δrate-phase transform’. This transform represents a relative encoding of relative input differences (detuning) between nearby neurons, irrespective of mean input levels. Changes in absolute input levels are in turn represented in the frequency of gamma oscillations. This is in line with the lack of rate-phase transform findings in the gamma range [45], with the finding of no phase-shifting with contrast [43] and the finding of phase-coding with orientation [34]. Orientation tuning is locally defined in visual cortex where nearby neurons are driven slightly differently by a given stimulus orientation. In this case, as shown in Tiesinga and Sejnowski [18], phase-coding should reflect the input differences between synchronized columns, independently however of overall input drive. We predict therefore that the gamma phase-coding of orientation should be insensitive to overall input strength, e.g. stimulus contrast.

**Link between coupling and phase coding**. According to the Arnold tongue, phase-relations between oscillating neurons are determined by input (intrinsic frequency) differences as well as coupling strength. This implies that for an exact interpretation of input differences, knowledge about the coupling values is required. In our reconstruction formula we multiplied the phase-differences with coupling values, such that phase-differences of more strongly coupled oscillators were weighted more strongly than from more weakly coupled oscillators. We conceptualize the included coupling term as a ‘prior’ (representing the general connectivity structure of a network) that might be used by the brain to optimize the input reconstruction performance based on phase. In general, we argue that the dependence of gamma phase coding on connectivity is of high interest and should be investigated in future studies, because it makes the code also sensitive to information (e.g. memory) imprinted in the network connectivity structure [60].

### Input-dependent spatial synchronization

We observed synchronization between nearby locations and the formation of gamma synchronization fields which were shaped by anatomical connectivity constraints and by the spatial pattern of input. Note that the input variable (excitatory drive) in the present paper has been mapped on visual contrast, but we expect to see the same network self-organization for stimulus features other than contrast, such as orientation and motion, which also modulate gamma frequency [42]. The use of visual contrast as an example parameter is related to the fact that contrast changes induce especially robust gamma frequency modulations [33,42,43], and that spatial changes in contrast have been demonstrated to lead localized differences in gamma frequency in nearby neural populations in visual cortex [43]. To our knowledge, this latter finding, although expected, has not been empirically demonstrated yet for sensory features other than visual contrast. In sum, we suggest that the input to the model can be mapped on many sensory variables.

In our model simulations, synchronization fields emerged in regions of high local input similarity (low detuning) where nearby neurons shared similar input properties. The shapes of those fields were input-specific; being small in regions of high local input variance and large in regions of low local variance. Their shapes were asymmetric around reference oscillators close to large discontinuities in input such as borders in a visual image. An analysis of the topographic network’s response to natural images showed that synchronization fields extended away from segmentation borders (as indicated by human observers), and did not cross them, in agreement with the network behavior described above. This network response matches with the statistics of natural images, in which segmentation borders are often associated with large local contrast changes [112], whereas the interior of surfaces often shows more modest variations in local contrast. This indicates that input-dependent spatial synchronization may be meaningful way to cluster/integrate nearby neurons based on input similarity.

The potential of oscillating neural networks for meaningful segmentation of input patterns has been well established in computational neuroscience studies [113–115] (in particular visual segmentation), which were inspired by experimental studies on stimulus-specific gamma synchronization over the last decades [2,21,30]. However, the proposed segmentation mechanisms differ between studies. In some studies, the clustering is based on a phase-code only [49,88,94,113], whereas in others it is mainly based on de-/synchronization [47]. Clustering capacity of a neuronal network model similar to the one proposed in our study has been demonstrated [116]. Our model architecture differs strongly from model networks characterized by global synchrony, like the LEGION model (local excitatory global inhibitory oscillator network, [88]) or the PCNN (pulse-coupled neural network, [113]), where clustering is based on phase alone and the network has a single main frequency at any given moment. LEGION and PCNN are powerful for image segmentation tasks, yet, they are not accurate models of cortical gamma oscillations, which are characterized by local synchrony and variable oscillation frequencies. However, they might be more appropriate model for slower oscillation phase [95] or latency coding [117]. In our simulations we did not explicitly investigate the clustering/segmentation performance of the neural network per se. However, our results give a new perspective into that matter that might guide future research in field of image segmentation. In particular, the TWCO offers a more precise understanding on how synchrony and phase in a self-organizing network relate to stimulus input characteristics and network connectivity. According to the Arnold tongue, input variations are transformed into both frequency (synchrony) and phase variations, and therefore both might be useful for clustering. We further suggest that clustering based on phase and synchrony/frequency would be complementary and represent fine and coarse spatial scales respectively. Finally, the Arnold tongue is an appropriate framework in linking clustering based on connectivity with clustering based on synchronization.

### Experimental predictions

From our theoretical analysis clear predictions can be derived that can be tested experimentally. We offer three key predictions:

1. For measuring the information content of gamma phase coding, e.g. during natural image stimulation in visual cortex, we predict that it is critical to measure the excitation *differences* between nearby locations, because we predict that gamma phase coding does not capture absolute input. We also expect that the frequency of gamma oscillations will contain a significant amount of information about the stimulus.
2. By manipulating both excitatory drive independently in two nearby cortical locations as well as the distance (coupling) between the locations, one should be able to reconstruct an Arnold tongue, assuming that cortical distance is correlated with a declining probability of connectivity. We expect that large excitation differences are expressed in oscillations frequency differences, whereas lower excitation differences will permit synchronization and a translation of the excitation differences into phase differences. This idea could be tested with optogenetic technology [118] by driving pyramidal cells with different intensities at different locations or by manipulating sensory stimulus features known to manipulate excitatory drive (e.g. contrast in V1).
3. Our ‘multiple oscillator model’ predicts that I-cells (e.g. FS) will also exhibit phase-differences, not just E-cells.

## Supporting Information

S1 Text Method S1 Text. Izhikevich-type neural network simulation.S1 Table. Parameter of Izhikevich network.S1 Code. Two Matlab simulation codes
Ring-PING network simulation with Izhikevich-type neurons.Ring-phase-oscillator network simulation

(DOC)

S1 Fig The effect of changing connection parameters in the ring-PING network. The main results are that we observed changes in the spatial synchronization properties as a function of different relative strengths of E-E, E-I, I-E and I-I synaptic connections. We present in this figure a heuristic for understanding these changes. In A) we show the two main types of connection in the PING networks, (I) the excitatory type (AMPA) and (II) the inhibitory (GABA-A) type. In B) we associate these two connection types with respective advancing (I) and delaying (II) part of the phase-response curve (PRC) assuming type 1 PRC [61]. Type 1 PRC means that excitatory synaptic input will advance the next spike (occurs earlier) and inhibitory synaptic input delays the next spike (occurs later). In C) the respective resultant Arnold tongues are depicted if only either excitatory (I) or inhibitory (II) would be present. In D) we show the resultant phase-locking matrix of the ring-phase-oscillator network simulation. In (I) we kept only an advancing component of the (infinitesimal) PRC, whereas in (II) only the delaying part. It can be observed that synchrony is enhanced around the peak of the sinusoidal input and reduced at the trough (I) and vice versa for (II). Advancing PRC (I) connections are good for entraining a lower frequency oscillation by a higher frequency oscillation which therefore leads to stronger synchronization around the higher frequencies of the sinusoidal input (peak). In E) we show the results of the ring PING network where we strengthened the dominance of excitatory connections (I) by either strengthening E-E connections (left) or reducing I-I connections (right). The phase-locking matrix resembled the matrix resulting from phase-oscillator simulations. In F) we strengthened the dominance of inhibitory connections by either decreasing E-E connections (left) or strengthening I-I connections. Again, the phase-locking matrix looks similar to the one with phase-oscillator networks with delaying PRC only.
(TIF)

S2 Fig The effect of noise on the ring-PING network. Main results are that noise reduced synchronization (shrinking Arnold tongue, see [18]) and dissociated single E-cell spike rate from the population gamma oscillation (as measured with LPA). Noise here was a Gaussian fluctuation of AMPA input rate over time added to the spatially defined sinusoidal mean input AMPA rate. *Top row*: A) Mean sinusoidal input (0.055±0.3×10^−2^mS/cm^2^) with low noise (SD = 0.9×10^−3^mS/cm^2^). B) The power spectra of the LPA contact points (see Methods) clearly showed gamma power with mainly two synchronous ‘components’ around the trough and peak of the sinusoidal input. In C) the phase-locking among all LPAs are shown in phase-locking matrix. In D) we quantified the gamma frequency (frequency of the maximal power peak). In E) the single E-cell spike rates are shown. Notice that D) and E) are highly similar. *Bottom row*: F) High noise input (SD = 5.6×10^−3^ mS/cm^2^). G) The power spectra of the LPAs still show gamma power with two main ‘components’, however reduced in size. In H) the LPA phase-locking matrix is shown. The synchronization between oscillators over larger distances is strongly reduced. In I) the gamma frequency is depicted still exhibiting frequency plateaus. The E-cell spikes rates in J) however lost these plateaus completely and have spike rates similar to the rates they would have if unconnected (intrinsic frequency).
(TIF)

S3 Fig Replication of Hodgkin-Huxley (HH) results in Fig. 3 using Izhikevich (IZ) neuron types (E-cell = regular-spiking type, I-cell = fast-spiking type). In the HH-network the E-cells had firings rates close to the gamma rhythm. However, pyramidal cells (E-cell) often have spikes rates lower than the gamma rhythm [14,15,92]. We therefore used IZ-type neurons with E-cells having lower rates than the gamma frequency. We used IZ-types because of their computational efficiency as it is known that low E-cell rates needs larger networks [85].(A) The general network architecture of the Izhi-network, replicating the architecture of main text Fig. 3, however with 80× more cells. (B) Extract of a simulation, where the black line (top) is a LPA signal, blue line is the voltage membrane of a I-cell (middle) and the red line is voltage membrane of an E-cell (bottom). Notice that the E-cell is skipping many gamma cycles. (C) Here the mean gamma frequency (black) estimated from the LPA, the mean E-cell spike rate (red) and the mean I-cell spike rate (blue) is shown. Whereas the I-cell had spike rates close to the gamma frequency (∼35–40Hz), the E-cell had spike rates much lower (∼10–15Hz). In D) the phase-locking (left) as well as the phase-relation (right) matrix is shown between all E-cell pairs in the matrix. The phase-relation values were threshold (0.3>) for illustrative reasons. The network behaved in similar manner as previously described even though individual E-cells had lower firing rates than the gamma rhythm.
(TIF)

S4 Fig Segmentation-border analysis. A) Capturing spatial windows (sized 31×1 pixels) for quantitative analysis illustrated in an example image. Windows were centered on a border and could be horizontal or vertical. All windows were then aligned to each other and concatenated. B) Mean phase-locking between all possible pairs of oscillators along border-centered spatial windows. Dashed line represents the 0-axis (position of the border). C) As for B, but phase-locking matrix was computed from spatial windows located randomly on the image to construct a null distribution. D) Differences in phase-locking between B and C, red colors indicate higher than expected phase-locking, blue lower than expected. White indicates that differences were not significant (permutation test, [77]).
(TIF)
