# Supplementary material for: Input-Dependent Frequency Modulation of Cortical Gamma Oscillations Shapes Spatial Synchronization and Enables Phase Coding
Source: PLoS Comput Biol. 2015 Feb 13;11(2):e1004072. doi: 10.1371/journal.pcbi.1004072 (PMC4334551; doi:10.1371/journal.pcbi.1004072)
Supplement: S1 Text — Method S1 Text. Izhikevich-type neural network simulation.S1 Table. Parameter of Izhikevich network.S1 Code. Two Matlab simulation codes Ring-PING network simulation with Izhikevich-type neurons.Ring-phase-oscillator network simulation (DOC) [file pcbi.1004072.s001.doc]

**Supporting Text S1**

**Method text S1**

**Izhikevich neural network.**

In Fig.3 the E-cells spiked regularly close to the network population rhythm. However, the E-cells’ spike rate is often reported to be lower than the gamma rhythm [15,90]. The minimum number of neurons needed to sustain stable gamma oscillations is higher for a sparsely firing network where E-cells fire only every second or third gamma cycle. To demonstrate that the same principles shown in Fig.3 also apply to more sparsely firing networks, we simulated (15sec) a ring gamma network with 4000 E-cells (regular-spiking type) and 1000 I-cells (fast-spiking type) based on an Izhikevich-type neuron model (IZ, [67]). Parameters are shown in Table S1.

$$\frac{dv}{dt}= 0.04v^{2}+5v+140-u+I$$

$$\frac{du}{dt}=a\left(bv-u\right)$$

$$if v \geq30mV, \text{then} \left\{\begin{matrix} v \leftarrow c \\ u \leftarrow u+d \end{matrix}\right.$$

Network input was similarly constructed as with the Hodgkin-Huxley type network with FS receiving external input current I=3.5mV (±SD=1.5) and RS a sinusoidal modulation of the mean input current 7mV (±SD=1.5) with a spatial sinusoidal amplitude of 1mV. The network connectivity was defined similarly to the phase-oscillator network model

$$\text{K}_{\text{i},\text{j}}=\text{C exp}^{-\text{D}_{\text{θ}}/\text{s}}\text{ }$$

where C is maximal connection strength, D is the distance matrix based on circular distance and s is the spatial scaling constant. Simulations based on connectivity structure as implemented in the Hodgkin-Huxley network revealed similar findings in terms of the phase-locking and phase-relation matrix (Fig.S3). The simulation MALTAB code for the IZ-type network as well as the ring phase-oscillator network) are provided in the addendum.

**Table S1. Parameter values of the Izhikevich-type network.**

| **parameter** | **Regular spiking neurons (RS)** | **Fast spiking interneuron (FS)** |
| --- | --- | --- |
| a | 0.02 | 0.1 |
| b | 0.2 | 0.2 |
| c (mV) | -65 | -65 |
| d | 8 | 2 |
| **Connectivity** | **Spatial constant s** | **Max connection strength (C )** |
| E🡪E | 0.4 | 0.004 |
| E🡪I | 0.3 | 0.07 |
| I🡪E | 0.3 | -0.04 |
| I🡪I | 0.3 | -0.015 |

*Upper half*: Neuron types are defined by the four parameter a, b, c and d in the Izhikevich neuron model [3]. These values are defined for regular spiking neurons (RS) in the middle column and for fast-spiking neurons (FS) in the right column. *Lower Half*: The connectivity matrix of the network. The spatial constant defines the rate of the exponential decay function of connectivity over space (middle column). The maximum connectivity strength is defined in the rightmost column.

**Code S1**

1. **The ring-Izhikevich-type PING network**

% Created by Eugene M. Izhikevich, February 25, 2003

% modified by E.Lowet, 5th September 2014

Ne=4000; Ni=1000; % number of excitatory and inhibitory neurons

a=[0.02*ones(Ne,1); 0.1*ones(Ni,1)]; % a =tiemscale of recover varibale u

b=[0.2*ones(Ne,1); 0.2*ones(Ni,1)]; % b= sensitivity of u to subthreshold oscillations

c=[-65*ones(Ne,1); -65*ones(Ni,1)]; % c= membrane voltage after spike (reset)

d=[8*ones(Ne,1); 2*ones(Ni,1)]; % d= spike reset of recover varibale u

v=-65*ones(Ne+Ni,1); % Initial values of v = voltage

u=b.*v; % Initial values of u= membrane recovery variable

firings=[]; % spike timings

simulation_time=8000 ;

dt=1;Ntot=(Ne+Ni);

disp('defining neurons done')

%%%%%%%%%%%%%% gaussian input%%%%%%%%%%%%%%%%%%%%%%%%%%%%%%

var_E= 1.5;%% to excitatory neurons

var_I= 1.5;%% to inhibitory neurons

%% creating main input stimulus

clear stim_input

Amplitude=1; % sinusoidal spatial modualtion of input strength

Meanlevel=7; % mean input level to RS cells

stim_input(1:Ne,1) = (sin((-(1*pi):(2*pi)/((Ne./1)-1):(1*pi)))).*Amplitude+Meanlevel;

stim_input(Ne+1:Ntot,1)= ones(Ni,1).*3.5; % additional mean inputto FS cells

%%%%%%%%%%%%%%%%%%%% synaptic constants %%%%%%%%%%%%%%%%%%%%%

gampa=zeros(Ne,1,'single');

gaba= zeros(Ni,1,'single');

decay_ampa =1;decay_gaba =4;

rise_ampa =0.1;rise_gaba =0.1;

%%%%%%%%%%%%%%%%%%%%%%%%%%%%%%%%%%%%%%%%%%%%%%%%%%%%%%%%%%%%

%% Constructing connectivity matrix %%%%%%%%%%%%%%%%%%%%%%%%

disp('start constructing connectivity matrix')

EE = 0.004;see=0.4; %% ecitatory to excitatory

EI = 0.07;sei=0.3; %% ecitatory to inhibitory

IE =-0.04;sie=0.3;%% inhibitory to excitatory

II =-0.015;sii=0.3; %% inhibitory to inhibitory

%%%%%%%%%%%%%%%%%%%%%%%%%%%%%%%%%%%%%%%%%%%%%%%%%%%%%%%%%%%%

S=zeros(Ntot,'single');dist=zeros(Ne,'single');

op=-pi:(2*pi)/(Ne-1):pi;

for ind=1:Ne

dist(ind,:) = abs( angle( exp(1i*op(ind) )./exp(1i*op)) );

end

dist((dist<0.001))= NaN; %

KEE= (EE*exp(-dist./see));

%%%%%%%%%%%%%%%%%%%%%%%%%%

dist=zeros(Ni,'single');

op=-pi:(2*pi)/(Ni-1):pi;

for ind=1:Ni

dist(ind,:) = abs( angle( exp(1i*op(ind) )./exp(1i*op)) );

end

dist((dist<0.001))= NaN;

KII= (II*exp(-dist./sii));

%%%%%%%%%%%%%%%%%%%%%%%%%%

dist=zeros(Ne,Ni,'single');

op2=-pi:(2*pi)/(Ne-1):pi;op=-pi:(2*pi)/(Ni-1):pi;

for ind=1:Ne

dist(ind,:) = abs( angle( exp(1i*op2(ind) )./exp(1i*op)) );

end

dist((dist<0.001))= NaN;

KEI= (EI*exp(-dist./sei));

%%%%%%%%%%%%%%%%%%%%%%

dist=zeros(Ni,Ne,'single');

op2=-pi:(2*pi)/(Ne-1):pi;op=-pi:(2*pi)/(Ni-1):pi;

for ind=1:Ni

dist(ind,:) =abs( angle( exp(1i*op(ind) )./exp(1i*op2)) );

end

dist((dist<0.001))= NaN;

KIE= (IE*exp(-dist./sie));

%%%%%%%%%%%%%%%%%%%%%%

S(1:Ne,1:Ne)= KEE;

S(Ne+1:Ntot,Ne+1:Ntot)= KII;

S(1:Ne,Ne+1:Ntot)= KIE';

S(Ne+1:Ntot,1:Ne)= KEI';

S(isnan(S))=0;S=single(S);

clear dist KII KIE KEI

disp('done constructing connectivity matrix')

%%%%%%%%%%%%%%%%%%%%%%%%%%%%%%%%%%%%%%%%%%%%%%%%%%%%%%%%%%%%%%%

%%%%%%%%%%%%%%%%%%%%%%%%%%%%%%%%%%%%%%%%%%%%%%%%%%%%%%%%%%%%%%%

%%%%%%%%%%% !!!!!! MAIN LOOP !!!!!!!! %%%%%%%%%%%%%%%%%%%%%%%%

disp('start simulation')

for t=1:dt:simulation_time

if mod(t,25) ==0

disp([ num2str(t) 'ms of ' num2str(simulation_time ) 'ms'])

end

I=[var_E*randn(Ne,1);var_I*randn(Ni,1)]+stim_input; % thalamic input

fired=find(v>=30); % indices of spikes

firings=[firings; t+0*fired,fired];

v(fired)=c(fired);

u(fired)=u(fired)+d(fired);

%synaptic potentials

gampa=gampa + dt*(0.3*(((1+tanh((v(1:Ne)/10) +2 ))/2).*(1-gampa)/rise_ampa - gampa/decay_ampa));

gaba= gaba + dt*(0.3*(((1+tanh((v(Ne+1:end)/10) +2 ))/2).*(1-gaba)/rise_gaba - gaba/decay_gaba));

gsyn=[gampa ;gaba];

% defining input to eah neuron as the summation of all synaptic input

% form all connected neurons

I=I+S*gsyn;

v=v+0.5*(0.04*v.^2+5*v+140-u+I); % step 0.5 ms

v=v+0.5*(0.04*v.^2+5*v+140-u+I); % for numerical

u=u+a.*(b.*v-u); % stability

end;

%%%%%%%%%%%%%%%%%%%%%%%%%%%%%%%%%%%%%%%%%%%%%%%%%%%%%%%%%%%%%%%

disp('done simulation')

%%%%%%%%%%%% *AFTER SIMULATION ANALYSIS* %%%%%%%%%%%%%%%%%%%%%%%

firings=int32(firings);

%%%%%%%%%%%%******** Plottting%%%%%%%%%%%%%%%%%%%%%%%%%%%%%%%%%%

disp('start plotting')

figure('Color','w','Position' ,[ 100 100 600 350]),

subplot(2,1,1,'Fontsize',15) % spike raster

firingexc=firings(find(firings(:,2) <=Ne),:);firinginh=firings(find(firings(:,2) > Ne),:);

plot(firingexc(:,1),firingexc(:,2),'.','Color', [ 0.8 0.2 0.2]); % spike raster

hold on, plot(firinginh(:,1),firinginh(:,2),'.','Color', [ 0.2 0.2 0.8]);

axis tight;set(gca,'xticklabel',[])

xlim([ 600 1800])

subplot(2,1,2,'Fontsize',15)

Fs = 1000./dt;[t1,t2] = hist(firings(:,1),0:1:t);

spectrogram(((t1)-mean(t1)),252,250,20:0.5:50,Fs,'Yaxis');

axis xy

xlim([ 0.6 1.8]);xlabel('Time s')

%%%%%%%%%%%%%%%%%%%%%%%%%%%%%%%%%%%%%%%%%%%%%%%%%%%%%%%%%%%%%%%%%%%%

%%%%% Making a spike matrix and computing spike rate %%%%%%%%%%%%%%%

clear spikerate spikerate2

rastersp=zeros(Ne,max(firingexc(:,1)),'int8');

rastersp2=zeros(Ni,max(firinginh(:,1)),'int8');

nn=0;

for ind= (1):(Ne) % RS

nn=nn+1;

rastersp(nn,firingexc(find(firingexc(:,2)==ind),1).*(1/dt))=1;

end

nn=0;

for ind= (Ne+1):(Ne+Ni) % FS

nn=nn+1;

rastersp2(nn,firinginh(find(firinginh(:,2)==ind),1).*(1/dt))=1;

end

%%%%%%%%%%%%%%%%%%%%%%%%%%%%%%%%%%%%%%%%%%%%%%%%%%%%%%%%%%%%%%%%%%%%%

%%%%%%%%%%%%%%%%%%%%%%%%%%%%%%%%%%%%%%%%%%%%%%%%%%%%%%%%%%%%%%%%%%%

disp('computation cross-correlatio matrix')

%%%%% Creating full phase-locking and phase difference matrix %%%%%%%%

spike_dat=rastersp2;

clear allcoh alltim

nn1=0;timwin=200:simulation_time-50;

for seed=(1:10:size(rastersp2,1))%steps of 10 to speed up

disp([num2str(seed) ' of ' num2str(size(rastersp2,1))])

nn1=nn1+1;nn2=0;

for ind=(1:10:size(rastersp2,1))%1

nn2=nn2+1;

if seed ~= ind

sig1= (double(spike_dat(seed:seed,timwin))'); %

sig2= (double(spike_dat(ind:ind,timwin))');

[c,lags]=xcorr( sig1 , sig2 ,12,'coeff'); % here +/- 12ms

[num1 num2]= max(c);

allcoh(nn1,nn2)= num1; %peak height

alltim(nn1,nn2)=num2; %peak lag

else % for the autocorrelation case

allcoh(nn1,nn2)= 1;

alltim(nn1,nn2)=13;

end

end

end

figure('COlor','w','Position',[300 300 240 200]),subplot(1,1,1,'Fontsize',15);

imagesc(allcoh); % spike cross-correlation peak

colormap('hot');%colorbar

set(gca,'CLim', [0 0.4])

set(gca,'xticklabel',[],'yticklabel',[]);

figure('COlor','w','Position',[300 300 240 200]),

phs=((alltim-13).*(-1));

for ind=1:size(phs,1)

for ind2=1:size(phs,2)

phs(ind,ind2)= phs(ind,ind2)./( (spikerate2(ind)+spikerate2(ind2))/2 ./2).*pi;%

end

end

subplot(1,1,1,'Fontsize',17);h=imagesc(phs); % spike timing difference

acoh=allcoh;

tt=(acoh)>0.1 & (acoh)<1; % phase-locking threshold (here arbitrarly defined)

set(h,'AlphaData',tt );

set(h, 'AlphaDataMapping', 'scaled');

set(gca,'xticklabel',[],'yticklabel',[]);

set(gca,'Clim',[-pi./2 pi./2])

1. **The ring-phase-oscillator network**

%modified Kuramoto model

% Eric Lowet,2013 ringe-network architecture with sinusodial intrinsic

% frequency variation

tic

clear all

number_of_oscillators=160; % --> increasing the oscillator number will increase the number of coupled oscillators...

initial_phase= (rand(number_of_oscillators,21)*1*(2*pi)); % Initial phases

simulation_time=5 %in sec

dt=0.002; %time step (here 2ms)

phases = zeros(number_of_oscillators,simulation_time./dt);

phases(:,1:21)= initial_phase;

%%%%Connectivity matrix %%%%%%%%%%%%%%%%%%%%%%%%%%%%%%%%%%%%%

clear dist

op=-pi:(2*pi)/(number_of_oscillators-1):pi;

for ind2=1:number_of_oscillators

dist(ind2,:) = abs( angle( exp(1i*op(ind2) )./exp(1i*op)) );

end

dist(dist<0.001) = NaN; % avoid connections with itself

s=0.4; %scaling constant for connectivity

C=1.65; % strength of connectivity

K= (C*exp(-dist./s));

%%%%%%%%%%%%%%%%%%%%%%%%%%%%%%%%%%%%%%%%%%%%%%%%%%

%%% DEFINING INTRINSIC FREQUENCIES%%%%%%%%%%%%%%%%%%%%%%%%%%%%%%%%%%%%%%%%%

Amplitude=3;

Mean_intrinsic_frequency =35;

W=(sin((-(1*pi):(2*pi)/((number_of_oscillators./1)-1):(1*pi)))).*Amplitude+Mean_intrinsic_frequency;

W=W.*(2*pi); %sum of radians per sec

%%%%%%%%%%%%%%%%%%%%%%%%%%%%%%%%%%%%%%%%%%%%%%%%%%%%%%%%%%%%%%%%%%%%%%%%%%%

clear noiseterm %%% pink noise (intrinsic/ dynamical noise)

for ind=1:number_of_oscillators

%%%%%%%%%%% from 2008.Little MA et al. (2007), "Exploiting nonlinear recurrence and fractal

% scaling properties for voice disorder detection", Biomed Eng Online, 6:23

N=(simulation_time./dt);alpha=1;

N2 = floor(N/2)-1;

f = (2:(N2+1))';

A2 = 1./(f.^(alpha/2));

p2 = (rand(N2,1)-0.5)*2*pi;

d2 = A2.*exp(i*p2);

d = [1; d2; 1/((N2+2)^alpha); flipud(conj(d2))];

x = real(ifft(d));

%%%%%%%%%%%%%%

noiseterm(ind,:)= ((x-mean(x))/std(x))./50; % phase detuning of std=0.02

end

%% noise is important to be able to distinguih true synchrony from false

%% synchrony (= insufficient dephasing)

%%%%%%%%%%%%%% MAIN LOOP %%%%%%%%%%%%%%%%%%%%%%%%%%%%%%%%%%%%%%%%%%%%%%%%%%

disp('start simulation')

for time=21:simulation_time./dt

if mod(time,50) ==0

disp([ num2str(time/(0.001/dt) ) 'ms of ' num2str(simulation_time*1000 ) 'ms'])

end

for ind=1:number_of_oscillators

interact=(sin((phases(ind,time-1) -phases(:,time-1)))) ;

phases(ind,time)= phases(ind,time-1) + (dt*W(ind)+ nansum(dt.*K(:,ind).* -interact ) ) + (noiseterm(ind,time));

end

end

disp('done simulation')

%%%%%%%%%%%%%%%%%%%%%%%%%%%%%%%%%%%%%%%%%%%%%%%%%%%%%%%%%%%%%%%%%%%%%%%%%%%

ph= (mod(phases',2*pi))'; %phases

xx=(exp(1i*ph(:,100:1:end))');

ab=xx' *xx;

allcoh=abs(ab);allcoh=allcoh./max(allcoh(:)); %phase locking matrix

allph=angle(ab); %phase relation matrix

%% Plotting the phase locking matrix

figure('COlor','w','Position',[300 300 300 200]),subplot(1,1,1,'Fontsize',17);imagesc(allcoh)

set(gca,'Clim',[ 0 1])

colormap('hot')

set(gca,'xticklabel',[],'yticklabel',[]);colorbar

axis xy

%% Plotting the phase relation matrix

coh_thres=0.25; % theshold for illustration

figure('COlor','w','Position',[640 300 300 200]),

subplot(1,1,1,'Fontsize',17);h=imagesc(allph)

acoh=allcoh;

tt=(acoh)>coh_thres; % phase-lcoking threshold

set(h,'AlphaData',tt );

set(h, 'AlphaDataMapping', 'scaled');

set(gca,'xticklabel',[],'yticklabel',[])

set(gca,'Clim',[ -pi/2 pi/2])

colorbar

axis xy

toc
