# Supplementary figures and images for: Input-Dependent Frequency Modulation of Cortical Gamma Oscillations Shapes Spatial Synchronization and Enables Phase Coding
Source: PLoS Comput Biol. 2015 Feb 13;11(2):e1004072. doi: 10.1371/journal.pcbi.1004072 (PMC4334551; doi:10.1371/journal.pcbi.1004072)

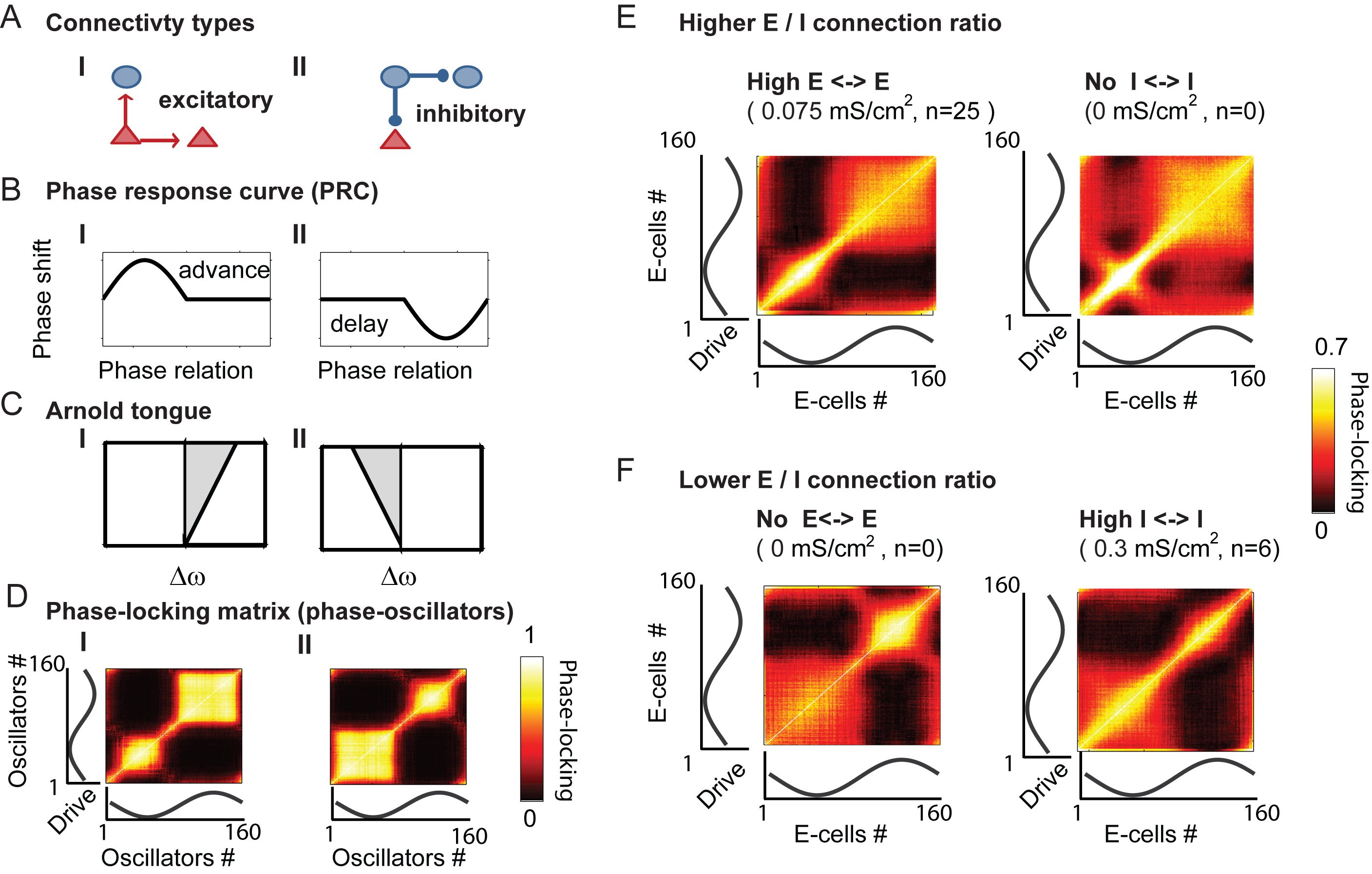

Supplement: S1 Fig — The main results are that we observed changes in the spatial synchronization properties as a function of different relative strengths of E-E, E-I, I-E and I-I synaptic connections. We present in this figure a heuristic for understanding these changes. In A) we show the two main types of connection in the PING networks, (I) the excitatory type (AMPA) and (II) the inhibitory (GABA-A) type. In B) we associate these two connection types with respective advancing (I) and delaying (II) part of the phase-response curve (PRC) assuming type 1 PRC [61]. Type 1 PRC means that excitatory synaptic input will advance the next spike (occurs earlier) and inhibitory synaptic input delays the next spike (occurs later). In C) the respective resultant Arnold tongues are depicted if only either excitatory (I) or inhibitory (II) would be present. In D) we show the resultant phase-locking matrix of the ring-phase-oscillator network simulation. In (I) we kept only an advancing component of the (infinitesimal) PRC, whereas in (II) only the delaying part. It can be observed that synchrony is enhanced around the peak of the sinusoidal input and reduced at the trough (I) and vice versa for (II). Advancing PRC (I) connections are good for entraining a lower frequency oscillation by a higher frequency oscillation which therefore leads to stronger synchronization around the higher frequencies of the sinusoidal input (peak). In E) we show the results of the ring PING network where we strengthened the dominance of excitatory connections (I) by either strengthening E-E connections (left) or reducing I-I connections (right). The phase-locking matrix resembled the matrix resulting from phase-oscillator simulations. In F) we strengthened the dominance of inhibitory connections by either decreasing E-E connections (left) or strengthening I-I connections. Again, the phase-locking matrix looks similar to the one with phase-oscillator networks with delaying PRC only. (TIF) [file pcbi.1004072.s002.tif]

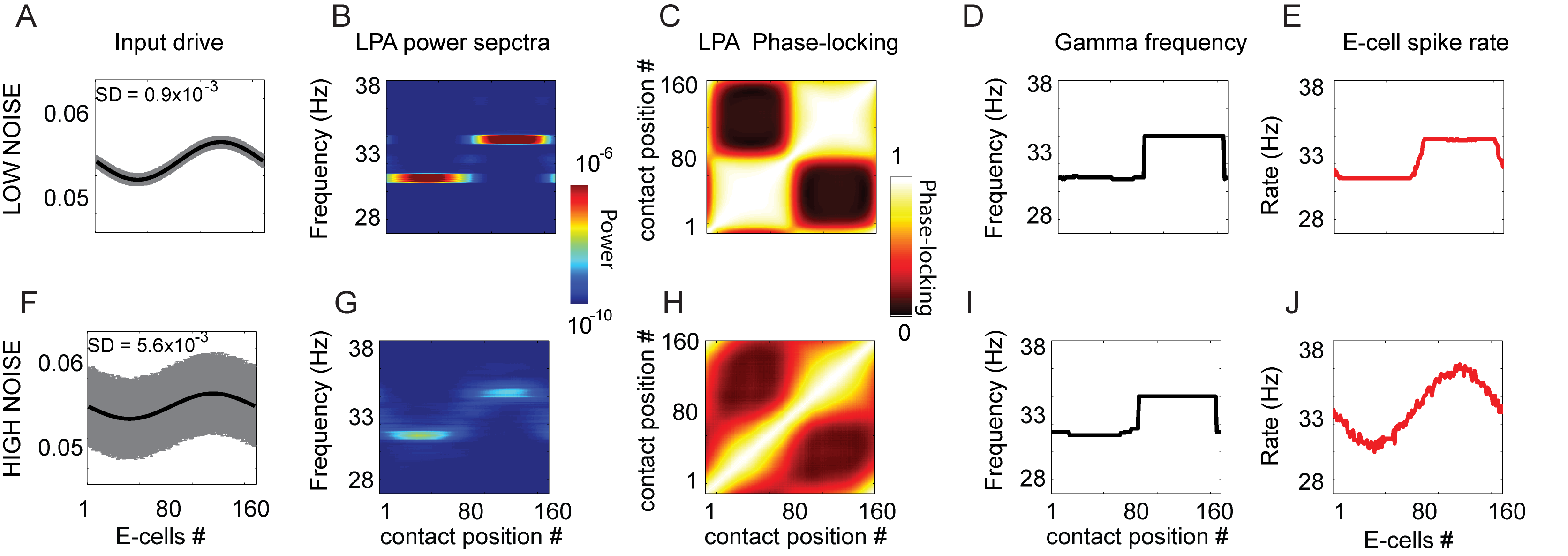

Supplement: S2 Fig — Main results are that noise reduced synchronization (shrinking Arnold tongue, see [18]) and dissociated single E-cell spike rate from the population gamma oscillation (as measured with LPA). Noise here was a Gaussian fluctuation of AMPA input rate over time added to the spatially defined sinusoidal mean input AMPA rate. Top row: A) Mean sinusoidal input (0.055±0.3×10^−2^mS/cm2) with low noise (SD = 0.9×10^−3^mS/cm2). B) The power spectra of the LPA contact points (see Methods) clearly showed gamma power with mainly two synchronous ‘components’ around the trough and peak of the sinusoidal input. In C) the phase-locking among all LPAs are shown in phase-locking matrix. In D) we quantified the gamma frequency (frequency of the maximal power peak). In E) the single E-cell spike rates are shown. Notice that D) and E) are highly similar. Bottom row: F) High noise input (SD = 5.6×10^−3^ mS/cm2). G) The power spectra of the LPAs still show gamma power with two main ‘components’, however reduced in size. In H) the LPA phase-locking matrix is shown. The synchronization between oscillators over larger distances is strongly reduced. In I) the gamma frequency is depicted still exhibiting frequency plateaus. The E-cell spikes rates in J) however lost these plateaus completely and have spike rates similar to the rates they would have if unconnected (intrinsic frequency). (TIF) [file pcbi.1004072.s003.tif]

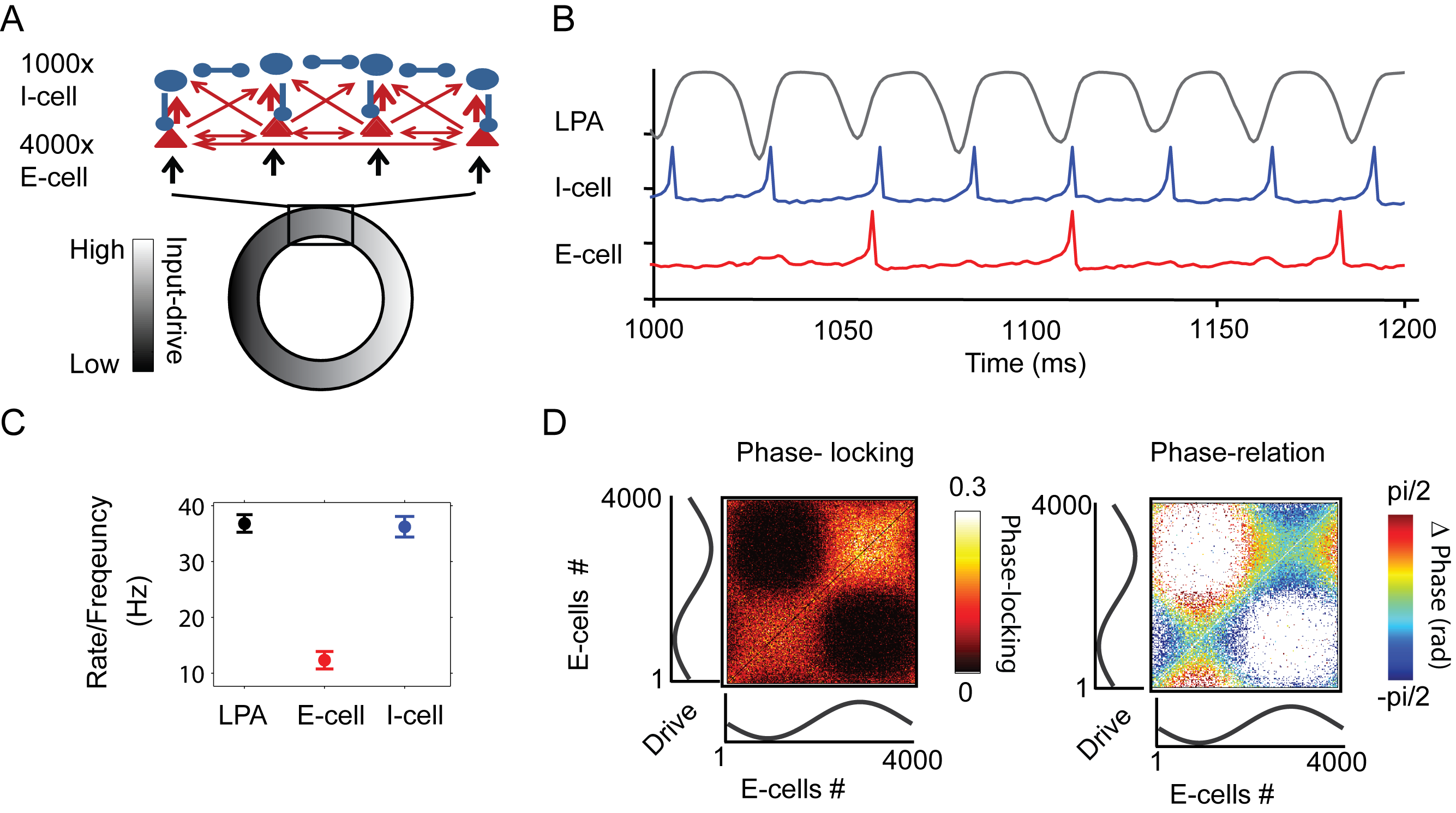

Supplement: S3 Fig — In the HH-network the E-cells had firings rates close to the gamma rhythm. However, pyramidal cells (E-cell) often have spikes rates lower than the gamma rhythm [14,15,92]. We therefore used IZ-type neurons with E-cells having lower rates than the gamma frequency. We used IZ-types because of their computational efficiency as it is known that low E-cell rates needs larger networks [85].(A) The general network architecture of the Izhi-network, replicating the architecture of main text Fig. 3, however with 80× more cells. (B) Extract of a simulation, where the black line (top) is a LPA signal, blue line is the voltage membrane of a I-cell (middle) and the red line is voltage membrane of an E-cell (bottom). Notice that the E-cell is skipping many gamma cycles. (C) Here the mean gamma frequency (black) estimated from the LPA, the mean E-cell spike rate (red) and the mean I-cell spike rate (blue) is shown. Whereas the I-cell had spike rates close to the gamma frequency (∼35–40Hz), the E-cell had spike rates much lower (∼10–15Hz). In D) the phase-locking (left) as well as the phase-relation (right) matrix is shown between all E-cell pairs in the matrix. The phase-relation values were threshold (0.3>) for illustrative reasons. The network behaved in similar manner as previously described even though individual E-cells had lower firing rates than the gamma rhythm. (TIF) [file pcbi.1004072.s004.tif]

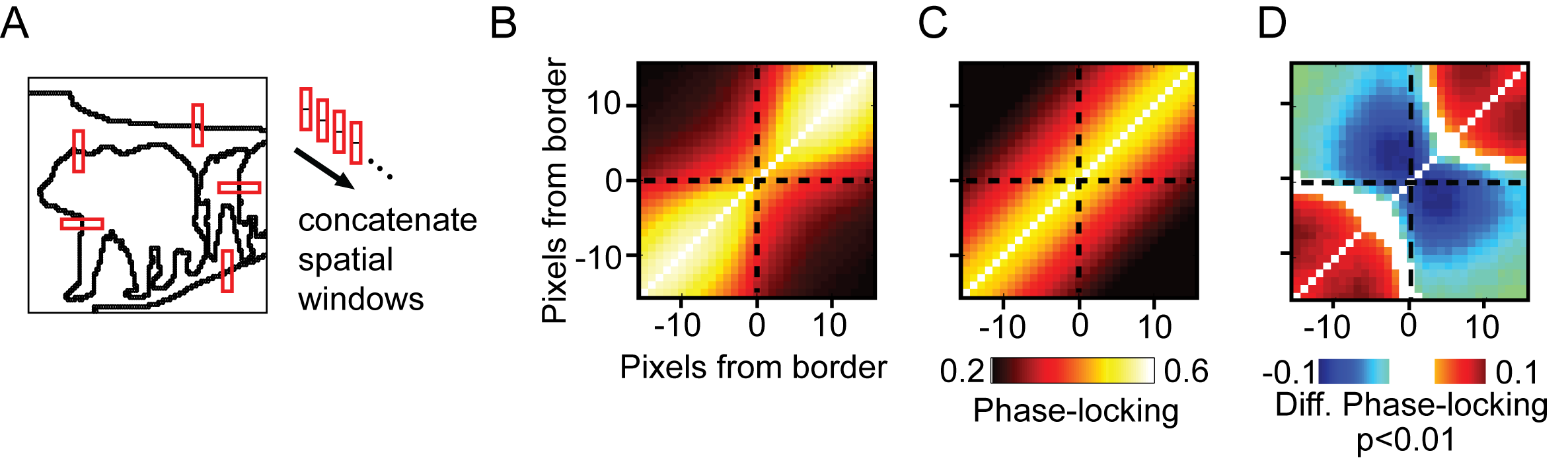

Supplement: S4 Fig — A) Capturing spatial windows (sized 31×1 pixels) for quantitative analysis illustrated in an example image. Windows were centered on a border and could be horizontal or vertical. All windows were then aligned to each other and concatenated. B) Mean phase-locking between all possible pairs of oscillators along border-centered spatial windows. Dashed line represents the 0-axis (position of the border). C) As for B, but phase-locking matrix was computed from spatial windows located randomly on the image to construct a null distribution. D) Differences in phase-locking between B and C, red colors indicate higher than expected phase-locking, blue lower than expected. White indicates that differences were not significant (permutation test, [77]). (TIF) [file pcbi.1004072.s005.tif]
